# Archaeal *Haloarcula californiae* Icosahedral Virus 1 Highlights Conserved Elements in Icosahedral Membrane-Containing DNA Viruses from Extreme Environments

**DOI:** 10.1128/mBio.00699-16

**Published:** 2016-07-19

**Authors:** Tatiana A. Demina, Maija K. Pietilä, Julija Svirskaitė, Janne J. Ravantti, Nina S. Atanasova, Dennis H. Bamford, Hanna M. Oksanen

**Affiliations:** Institute of Biotechnology and Department of Biosciences, University of Helsinki, Helsinki, Finland

## Abstract

Despite their high genomic diversity, all known viruses are structurally constrained to a limited number of virion morphotypes. One morphotype of viruses infecting bacteria, archaea, and eukaryotes is the tailless icosahedral morphotype with an internal membrane. Although it is considered an abundant morphotype in extreme environments, only seven such archaeal viruses are known. Here, we introduce *Haloarcula californiae* icosahedral virus 1 (HCIV-1), a halophilic euryarchaeal virus originating from salt crystals. HCIV-1 also retains its infectivity under low-salinity conditions, showing that it is able to adapt to environmental changes. The release of progeny virions resulting from cell lysis was evidenced by reduced cellular oxygen consumption, leakage of intracellular ATP, and binding of an indicator ion to ruptured cell membranes. The virion contains at least 12 different protein species, lipids selectively acquired from the host cell membrane, and a 31,314-bp-long linear double-stranded DNA (dsDNA). The overall genome organization and sequence show high similarity to the genomes of archaeal viruses in the *Sphaerolipoviridae* family. Phylogenetic analysis based on the major conserved components needed for virion assembly—the major capsid proteins and the packaging ATPase—placed HCIV-1 along with the alphasphaerolipoviruses in a distinct, well-supported clade. On the basis of its virion morphology and sequence similarities, most notably, those of its core virion components, we propose that HCIV-1 is a member of the PRD1-adenovirus structure-based lineage together with other sphaerolipoviruses. This addition to the lineage reinforces the notion of the ancient evolutionary links observed between the viruses and further highlights the limits of the choices found in nature for formation of a virion.

## IMPORTANCE

Under conditions of extreme salinity, the majority of the organisms present are archaea, which encounter substantial selective pressure, being constantly attacked by viruses. Regardless of the enormous viral sequence diversity, all known viruses can be clustered into a few structure-based viral lineages based on their core virion components. Our description of a new halophilic virus-host system adds significant insights into the largely unstudied field of archaeal viruses and, in general, of life under extreme conditions. Comprehensive molecular characterization of HCIV-1 shows that this icosahedral internal membrane-containing virus exhibits conserved elements responsible for virion organization. This places the virus neatly in the PRD1-adenovirus structure-based lineage. HCIV-1 further highlights the limited diversity of virus morphotypes despite the astronomical number of viruses in the biosphere. The observed high conservation in the core virion elements should be considered in addressing such fundamental issues as the origin and evolution of viruses and their interplay with their hosts.

## INTRODUCTION

*Archaea* dominate in extreme environments; consequently, ~140 known archaeal viruses have been isolated, mainly from geothermal springs and high-salinity environments (1). Viruses infecting crenarchea are morphologically diverse, whereas most of the known euryarchaeal viruses resemble tailed icosahedral bacteriophages (1). Pleomorphic, spindle-shaped, and tailless icosahedral euryarchaeal viruses have also been described (1). Of particular interest are the tailless icosahedral viruses that contain an internal membrane. These viruses infect crenarchea, euryarchaea, bacteria, or eukaryotes, and PRD1 serves for them as a model (2). At present, seven such archaeal viruses are known: thermophilic STIV (*Sulfolobus* turreted icosahedral virus) (3) and STIV2 (*Sulfolobus* turreted icosahedral virus 2) (4) as well as halophilic SH1 (5–7), SNJ1 (8), HHIV-2 (*Haloarcula hispanica* icosahedral virus 2) (9), PH1 (10), and the most recent addition, HCIV-1 (*Haloarcula californiae* icosahedral virus 1) (11).

According to the hypothesis of viral structural lineages, the evolutionary relationships between different viral groups can be resolved by comparing virion structures (12, 13). Despite the vast genomic diversity of viruses, all viruses known today represent a very limited number of morphotypes. Each virus structural lineage comprises viruses sharing the same major capsid protein (MCP) fold and virion architecture, and so far, four lineages have been established (14). One of these is the PRD1-adenovirus lineage comprising icosahedral, double-stranded DNA (dsDNA) viruses that infect hosts from all three domains of cellular life (14). These viruses share the canonical upright double β-barrel MCP fold (2) (also called vertical double β-barrel viruses) and a packaging ATPase with conserved motifs (15). The high-resolution structure of the STIV MCP places the virus within this lineage (12, 14) along with bacteriophages PRD1 (2) and PM2 (16), eukaryotic adenovirus (17), and chlorella virus PBCV-1 (*Paramecium bursaria* chlorella virus 1) (18) as well as others. Recently, a group of PRD1-adenovirus-like viruses with two MCPs instead of one, known as the vertical single β-barrel viruses, has been identified. This group comprises bacteriophages P23-77 (19), IN93 (20), and SSIP-1 (*Salisaeta* icosahedral phage 1) (21) and several proviruses (10, 22, 23) as well as SH1 (5), SNJ1 (8), HHIV-2 (9, 24), and PH1 (10). High-resolution structures of the MCPs of P23-77 are available: the small MCP VP16 (virion protein 16) is a vertical single β-barrel, while the large MCP VP17 consists of two vertical single β-barrel domains stacked one atop the other (19). VP16 homodimers and VP17 monomers assemble together to form the pseudohexameric capsomers arranged in the capsid lattice with a pseudotriangulation number (*T*) of T=28 (19). Two other viruses known to have similar capsid geometries are archaeal viruses SH1 and HHIV-2 (see below) (5, 24).

SH1 is now the best studied tailless icosahedral virus with an internal membrane that infects halophilic archaea (5–7). The SH1 capsid (~80 nm in diameter) is organized with a lattice with T=28, as is that of bacteriophage P23-77 (5, 19). There are a total of ~15 virion structural proteins, including the two MCPs VP4 and VP7. The folds of both MCPs are most probably vertical single β-barrels (5, 7). Proteins VP2, VP3, and VP6 (and perhaps others) comprise the vertex complex used for host recognition (5). The major membrane proteins VP10 and VP12 (25) and several structural protein species are associated with an internal membrane that consists of lipids selectively acquired from the host lipid membrane (7, 25). The linear 30,898-bp dsDNA genome contains 56 open reading frames (ORFs) (7). The inverted terminal repeats and terminal proteins suggest that SH1 uses a protein-primed mode of replication (26). Progeny viruses are released via host cell lysis (6, 27).

Like SH1, *Haloarcula hispanica* viruses HHIV-2 and PH1 (9, 10) are virulent viruses with narrow host ranges and similar virion morphologies (9, 10). All three share similar organizations of their linear dsDNA genomes, as well as several homologous genes (7, 9, 10). A recent study of the HHIV-2 virion revealed a pseudocapsid structure corresponding to T=28, like that of SH1 (24). However, instead of the horn-shaped host recognition complexes of SH1, HHIV-2 has propeller-like spike complexes with a flexible fiber at the 5-fold positions (24). In spite of that, SH1 and HHIV-2 have rather similar host ranges (6, 9). SH1, HHIV-2, and PH1 have been assigned to the *Sphaerolipoviridae* family (*Alphasphaerolipovirus* genus). The SNJ1 virus of *Natrinema* also belongs to the *Sphaerolipoviridae* family. It is a temperate virus with a plasmid prophage (8) that switches between virulent and temperate replication modes depending on the salt concentration (28). Furthermore, there is no synteny between the circular SNJ1 genome (~16 kb) and the genomes of other sphaerolipoviruses (8). Consequently, SNJ1 was assigned to the *Betasphaerolipovirus* genus.

During our search for new halophilic archaeal viruses, we isolated HCIV-1, a tailless icosahedral virus from salt crystals collected from the Samut Sakhon solar saltern in Thailand that infects *Haloarcula californiae* (11). The host range of HCIV-1 is not restricted to its isolation host but also includes *Haloarcula japonica*, *Halorubrum* sp. strain SS7-4, and *Haloarcula hispanica* (11). Here we report the detailed characterization of HCIV-1. We show that HCIV-1 is stable under various conditions and that virus infection results in host cell lysis with high virus production. We also demonstrate that HCIV-1 is a membrane-containing virus whose genome sequence and structural proteins reveal its close relationship to the alphasphaerolipoviruses.

## RESULTS

### Archaeal virus HCIV-1 isolated from an extreme environment tolerates a wide range of salinities.

HCIV-1 was recognized to be a potential model virus because high-titer agar stocks (typically ~2 × 10^11^ PFU/ml) were stable for at least 4 weeks at 4°C. Although HCIV-1 originated from high-salinity conditions, it tolerates high ionic strength (up to 23% salt water [SW] containing 3.15 M NaCl) as well as low-ionic-strength environments. Its infectivity decreased only at the lowest salinities tested (1.8% SW and 0.7% SW, containing 250 mM and 96 mM NaCl, respectively; see Fig. S1A in the supplemental material). When only NaCl (0.05 to 3.15 M) or magnesium ion (5 to 200 mM) concentrations were adjusted, no major changes in infectivity were detected (see Fig. S1B and C). In addition, removal of the calcium ions had no significant effect on infectivity. HCIV-1 preserved its infectivity in the designed HCIV-1 buffer with a total salinity level of ~6.6% for the 2-week period of testing (see Fig. S1D). HCIV-1 also tolerated temperatures up to 60°C (see Fig. S1E) and stayed infectious over a pH range of 5.5 to 9.0 (see Fig. S1F).

### HCIV-1 progeny viruses exit the euryarchaeal cells by disrupting the cell membrane envelope.

To optimize HCIV-1 production in liquid culture, *Haloarcula californiae* cells in different growth phases (early, middle, and late exponential) were infected using a multiplicity of infection (MOI) of 25. The highest virus production (~1 × 10^10^ PFU/ml in culture supernatants) was obtained when cells were infected during mid-exponential-phase growth. Varying the MOI from 3 to 30 had no significant effect on virus production (8 × 10^9^ to 1 × 10^10^ PFU/ml). Consequently, virus production and life cycle studies were carried out using cells infected at a MOI of 10 in mid-exponential-phase growth.

**FIG 1:**
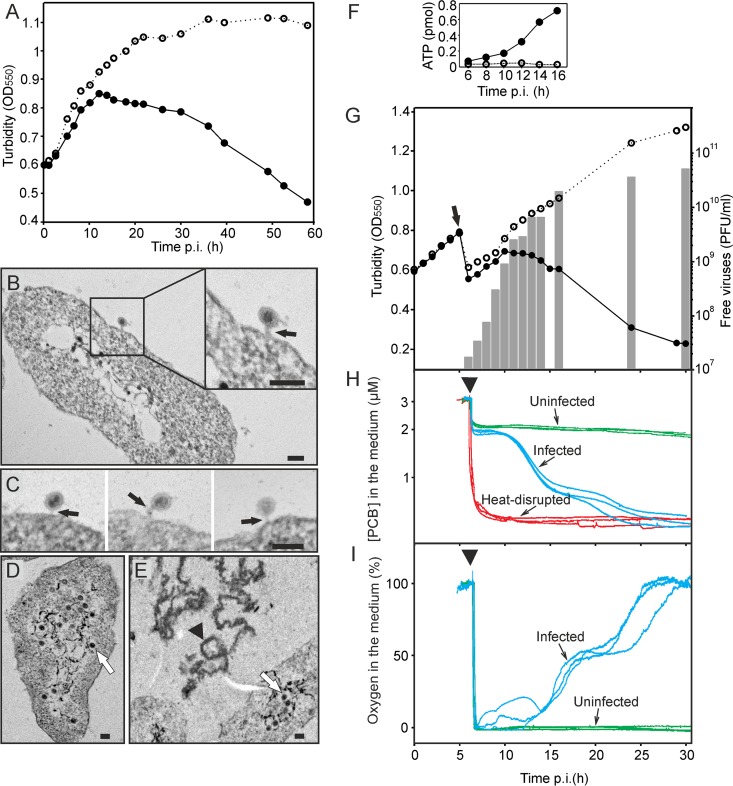
HCIV-1 infection cycle. (A) Growth curves of uninfected (open circles) and HCIV-1-infected (closed circles) *Haloarcula californiae* cultures. (B to E) Thin-section electron micrographs of HCIV-1-infected cells. (B and C) Virus particles attached to the cells at 5 h p.i. (black arrows) with tube-like structures between the viral particles and cells. (D) Intracellular virus particles at 8 h p.i. (white arrow). (E) Intracellular virus particles (white arrow) and cell debris (black arrowhead) indicating cell lysis at 12 h p.i. Scale bars in panels B to E, 100 nm. (F) Amount of extracellular ATP in the infected (closed circles) and uninfected (open circles) *Haloarcula californiae* cultures. (G) Growth curves of uninfected (open circles) and HCIV-1-infected (closed circles) *Haloarcula californiae* cells and the number of free progeny viruses in the infected culture (bars). The time point (5 h p.i.; arrow) when the cultures were washed to remove unadsorbed virus particles is indicated. (H) Binding of PCB^–^ to infected (blue lines), uninfected (green lines), and heat-disrupted (red lines) *Haloarcula californiae* cells measured starting at ~6 h p.i. (arrowhead) (*n* = 3) in the presence of PCB^−^ (calibration was performed with 3 μM PCB^–^). (I) The relative concentrations (%) of dissolved oxygen in the medium of infected (blue lines) and uninfected (green lines) cultures measured starting at ~6 h p.i. (arrowhead). The MOI was 60 for the experiments represented in panels B and C; elsewhere, the MOI was 10. The cells were grown in flasks (A to E) or reaction vessels (F to I) at 37°C with aeration.

The adsorption of HCIV-1 virions to the cell surface of *Haloarcula californiae* was efficient but rather slow, with an adsorption rate constant of 5.7 × 10^−11^ ml/min (calculated during the first hour postinfection [p.i.]). At 4 to 5 h p.i., ~80% of the particles were adsorbed to cells (see Fig. S2A in the supplemental material). A typical one-step growth curve of HCIV-1 infection showed a drop in turbidity indicating cell lysis at ~12 h p.i. (Fig. 1A). The adsorption experiment gave the same time point for the release of progeny viruses (12 h p.i.; see Fig. S2A). The burst size was ~100 progeny viruses per infected cell. That the number of infective centers approximately equaled the number of viable cells by 2 to 3 h p.i. (~1.3 × 10^8^) demonstrated efficient infection. Transmission electron microscopy (TEM) of infected cells showed viruses adsorbed on the host cells at 2.5 h p.i. (Fig. 1B and C), but genome delivery had not yet occurred, as indicated by the absence of empty capsids. A few viruses were attached to the cell surface by a tube-like appendage (Fig. 1B and C). The first assembled virions were observed inside the cells at 5 to 6 h p.i. (Fig. 1D), and cell debris was visible at 12 h p.i. (Fig. 1E).

To determine whether HCIV-1 progeny release involved cell lysis, we used several techniques to monitor changes in *Haloarcula californiae* physiology. We measured the changes in cell integrity using the lipophilic indicator anion phenyldicarbaundecaborane (PCB^−^) and also by monitoring cellular oxygen consumption and the release of ATP from the cells (Fig. 1F to I). Leakage of ATP indicating openings in the cell envelope was detectable at the start of the major virus release period at 8 to 10 h p.i., followed by the decrease in turbidity of the infected culture at 10 to 12 h p.i. (Fig. 1F and G). Uninfected cells did not bind PCB^−^ during the experiments, whereas heat-disrupted cells rapidly accumulated significant amounts of PCB^−^ (Fig. 1H). The increase in the number of progeny viruses was concurrent with increased PCB^−^ binding, revealing loss of cell envelope integrity (Fig. 1G and H), and also with the decreased oxygen level, indicating compromised respiration (Fig. 1I). The first cell debris was also observed at ~8 h p.i. (see Fig. S2B in the supplemental material). On the basis of all these data, we concluded that HCIV-1 virion release occurs via cell lysis.

### The complex HCIV-1 virion is composed of lipids, proteins, and nucleic acid.

**FIG 2:**
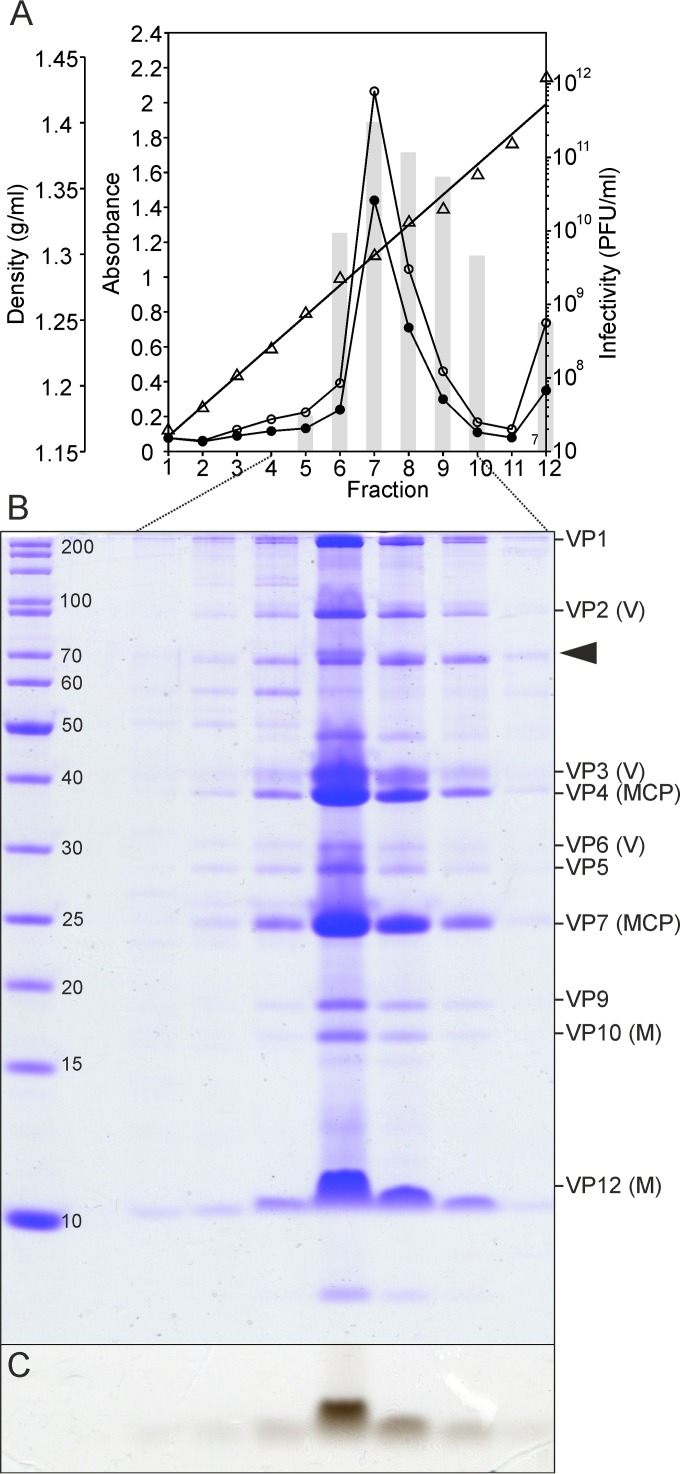
Protein and lipid content of HCIV-1 virions equilibrated in CsCl. (A) Density (triangles), infectivity (bars), *A*_260_ values (open circles), and *A*_280_ values (closed circles) of the CsCl gradient fractions. (B) Proteins from fractions 4 to 10 separated in polyacrylamide-Tricine-SDS gel stained with Coomassie blue. Molecular mass markers (in kilodaltons) (left lane) and HCIV-1 structural proteins (right lane) are shown. Virion proteins (VPs) and their predicted functions are indicated on the right (M, membrane proteins; MCP, major capsid proteins; V, vertex proteins). One host-derived impurity was identified by mass spectrometry (arrowhead). (C) Lipid signal detected below the 10-kDa protein marker after staining the separation gel with Sudan Black B.

**FIG 3:**
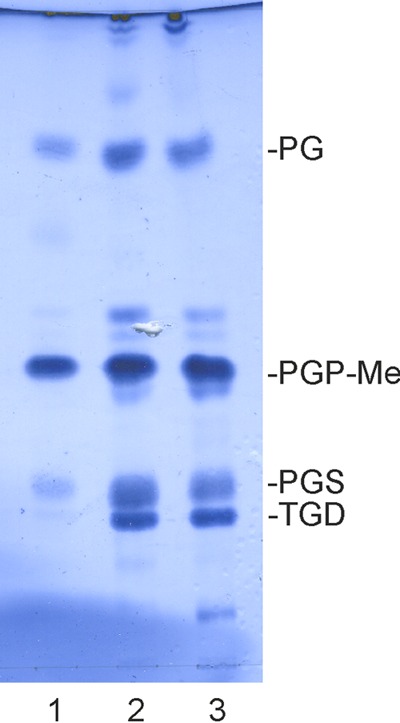
HCIV-1 virion lipids. A thin-layer chromatogram of lipids extracted from the twice-purified HCIV-1 virions (lane 1), *Haloarcula californiae* cells (lane 2), and *Haloarcula hispanica* cells (lane 3) is shown. The major lipid species of *Haloarcula hispanica* are indicated on the right (7) as follows: PG, phosphatidylglycerol; PGP-Me, phosphatidylglycerophosphate methyl ester; PGS, phosphatidylglycerosulfate; TGD, triglycosyl glycerodiether.

We optimized a multistep purification procedure for HCIV-1 that yielded particles of near-homogeneity with a specific infectivity of ~1.4 × 10^12^ PFU/mg of protein for the twice-purified HCIV-1 (see Table S1 in the supplemental material), which is comparable to the specific infectivity of highly purified SH1 (25). The final yield was ~1.7 mg of twice-purified HCIV-1 from 1 liter of lysate, and the rate of recovery of infectious particles was ~17% (see Table S1). The equilibrated infectious zone in the CsCl gradient (~1.30 g/ml; Fig. 2A) contained a complex set of proteins (Fig. 2A and B), as well as a lipid signal detected by Sudan Black B staining (Fig. 2C). Both the lower infectivity in the presence of chloroform (11) and the low virion buoyant density also suggested the presence of lipids in the virion. Viral lipids were extracted from the twice-purified virions and analyzed by thin-layer chromatography. The lipid profile of HCIV-1 differed from that of *Haloarcula californiae*, indicating selective acquisition of host membrane lipids by HCIV-1 (Fig. 3). Comparison with the known *Haloarcula hispanica* lipid species (7) identified the major HCIV-1 phospholipids as phosphatidyl glycerol, phosphatidyl glycerophosphate methyl ester, and phosphatidyl glycerosulphate (Fig. 3).

**FIG 4:**
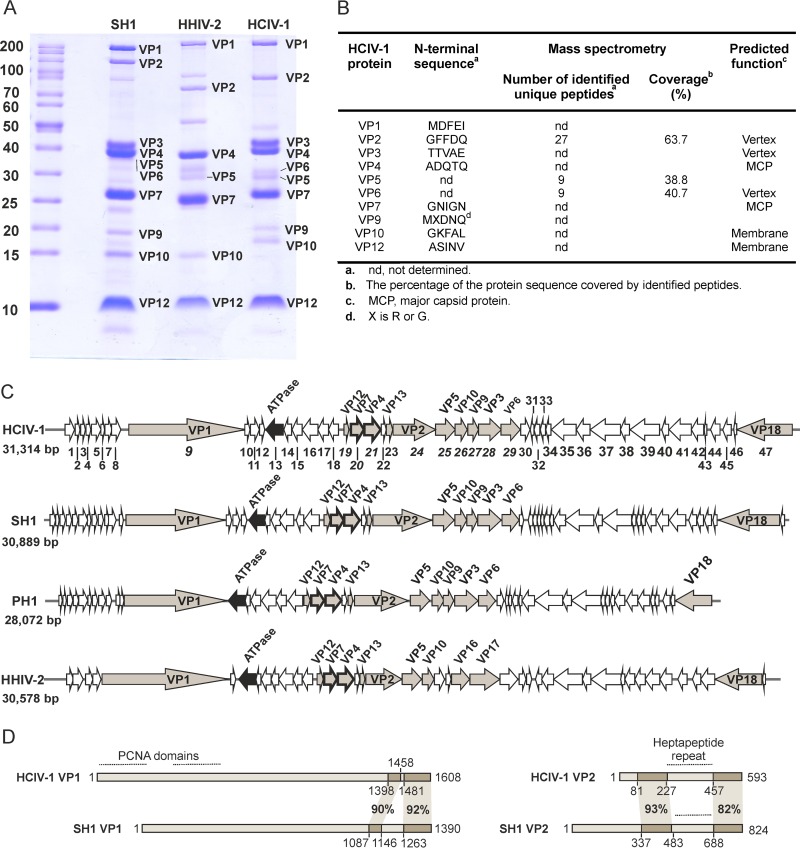
HCIV-1 structural proteins and genome organization. (A) Virion proteins of highly purified SH1, HHIV-2, and HCIV-1 analyzed in a polyacrylamide-Tricine-SDS gel stained with Coomassie blue. Molecular mass markers are indicated in kilodaltons (left lane). (B) Identification of HCIV-1 structural proteins by N-terminal sequencing and mass spectrometry. (C) Comparison of the HCIV-1, SH1, PH1, and HHIV-2 genomes. The reading direction of genes/ORFs is indicated (arrows), and HCIV-1 gene/ORF numbers (1 to 47) are shown; also indicated are genes/ORFs encoding VPs (grey), putative ATPases (black), and MCPs VP4 and VP7 (thicker lines). Genes encoding structural proteins are labeled as VPs. (D) Comparison of proteins VP1 and VP2 of HCIV-1 and SH1. Amino acid coordinates and similarities (%) for conserved regions are shown. Proliferating cell nuclear antigen (PCNA) domains in HCIV-1 VP1, as well as heptapeptide regions in HCIV-1 and SH1 VP2 proteins, are highlighted with lines.

**FIG 5:**
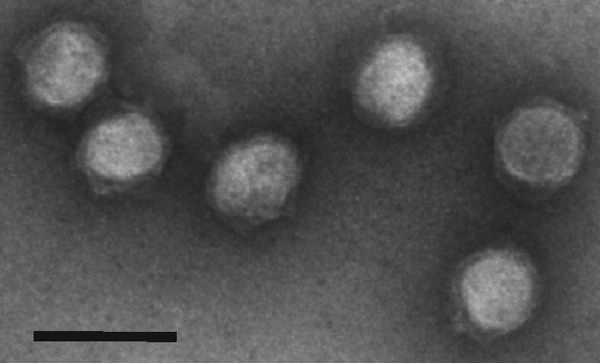
TEM micrographs of the twice-purified HCIV-1 virions stained with 3% (wt/vol) uranyl acetate (pH 4.5). Scale bar, 100 nm.

The HCIV-1 virion is composed of at least 12 protein species (Fig. 4A and B), which were numbered according to their similarities to the corresponding proteins of SH1 and HHIV-2 (see Table S2 in the supplemental material). The N-terminal methionine had been cleaved from all detected N-terminal sequences, except those of VP1 and VP9. None of the HCIV-1 structural proteins were shown to be glycosylated (see Fig. S3). Overall, the structural protein profile of HCIV-1 was definitely similar, but not identical, to those of SH1 and HHIV-2 (Fig. 4A). HCIV-1 virions appeared as tailless icosahedrons, ~70 nm in diameter, with an inner layer (membrane) visible beneath the protein capsid (Fig. 5).

### The HCIV-1 genome is a linear dsDNA molecule.

The HCIV-1 nucleic acid was degraded by RQ1 DNase and Exonuclease III, but not by Mung Bean nuclease or RNase A, and was cleaved by restriction enzymes AseI, MseI, SmaI, and HincII, suggesting that the genome is a linear dsDNA molecule. Sequencing of the molecule revealed that the genome is a linear dsDNA of 31,314 bp (see below for the NCBI accession number). The GC content is high (~68%), which is typical for halophilic organisms, and there are short inverted terminal repeats in the genome ends (19 bp; CATCTCTCTCTCTCTCTCT). Comparison of the levels of mobility during agarose gel electrophoresis of HCIV-1 nucleic acids extracted with and without protease treatment suggested the possible attachment of terminal proteins to the DNA ends (data not shown) (9, 10, 29).

A total of 47 ORFs, possibly coding for peptides greater than 38 amino acids in length, were identified. The ORFs are tightly packed in the genome (1.5 ORFs/kb) (Fig. 4C; see also Table S2 in the supplemental material). The majority of the proteins have a low calculated isoelectric point (pI, <5) typical of haloarchaeal proteins (30). Ten ORFs were confirmed to be genes encoding the following virion proteins: VP1 to VP7, VP9, VP10, and VP12 (Fig. 4A and B). On the basis of the similarities with SH1 and HHIV-2 structural proteins and their known positions in the virions (5, 24), HCIV-1 proteins VP4 and VP7 are suggested to be the two MCPs, while proteins VP2, VP3, and VP6 form the vertex complex, and both VP10 and VP12 are most probably located in the membrane (Fig. 4B).

A BLAST search found that 43 of the 47 HCIV-1 (putative) proteins had matches to haloarchaeal viral sequences (see Table S2 in the supplemental material) (threshold of 25% amino acid similarity) or to archaeal proteins (see below). Only putative proteins 3, 6, 32, and 34 had no significant similarity to any sequence in the database. Considering the high pI values calculated for the predicted products of ORFs 3 and 6 (see Table S2), it is uncertain whether these ORFs are encoding regions. Most of the matching viral sequences were from alphasphaerolipoviruses SH1, HHIV-2, and PH1 (see below and Table S2). Several matches to other haloarchaeal viral sequences, including those of tailed icosahedral, lemon-shaped, and pleomorphic viruses, were also found, as well as some hits to bacteriophage sequences with unknown functions (see Table S2). However, these hits did not give any significant information about HCIV-1 protein functions. HCIV-1 putative protein 1 showed ~40% similarity to putative proteins of five tailed icosahedral haloarchaeal *Halorubrum* viruses: HSTV-2, HRTV-7, HRTV-5, HRTV-8, and HF2 (see Table S2). Interestingly, all five of these hypothetical proteins are encoded by ORFs that are followed by a tRNA-coding region(s) (Gln, Arg, or Asn), except for HRTV-5, where two ORFs separate ORF 78 from the tRNA-coding ORF. However, no tRNA-coding elements were found close to HCIV-1 ORF 1. The only putative tRNA-Gln (CTG) coding sequence was in a downstream region within ORFs 46 and 47 (coordinates 28,559 to 28,771 in the genome; complement) with a 128-bp intron.

Tandem repeats were identified in ORFs 16 and 47, gene *24* (encoding VP2), and at the right end of the genome (upstream of ORF 47) (see Table S3 in the supplemental material). We identified a heptapeptide repeat pattern in HCIV-1 VP2 (Fig. 4D; see also Fig. S4 in the supplemental material) similar to the previously reported ones in the VP2 proteins of SH1 (7), HHIV-2 (9), and PH1 (10). The heptapeptide repeat pattern is characteristic of coiled-coil proteins, suggesting that VP2 proteins could be elongated fiber-like proteins. In addition to HCIV-1 VP2, the coiled-coil regions were found in 11 (putative) HCIV-1 proteins (see Table S2). The only structural protein species of HCIV-1 with predicted transmembrane helices is VP12, suggesting that it is tightly embedded in the membrane. There were also five other putative proteins with predicted transmembrane helices (see Table S2).

Conserved domains were identified in HCIV-1 VP1 (Fig. 4D) and VP5 and in putative proteins 13, 32, 33, 34, 41, 42, 44, and 46 (see Table S4 in the supplemental material). VP1 is predicted to contain two proliferating cell nuclear antigen (PCNA) domains (Fig. 4D; see also Table S4) that are similar to the DNA polymerase sliding clamps of various haloarchaeal species, including *Haloarcula californiae* and *Haloarcula hispanica*. However, we were not able to identify any ORF with canonical DNA polymerase motifs, suggesting that HCIV-1 uses its host cell replication machinery. Putative protein 44 was predicted to have an RNA polymerase sigma subunit domain similar to the transcriptional regulators of archaea and bacteria. ORF 13 is predicted to encode a packaging ATPase based on sequence similarity to the putative packaging ATPases of haloviruses SH1, HHIV-2, and PH1 (see Table S2) and also based on the presence of an AAA-like domain (see Table S4). This putative ATPase contains the Walker A and B motifs, as well as the P9/A32-specific conserved sequence, which is also present in the PRD1 packaging ATPase P9 (15) and in the putative packaging ATPases of other membrane-containing icosahedral viruses such as Bam35, PM2, SH1, and STIV (15). In icosahedral membrane-containing viruses such as PRD1 and PM2, the packaging ATPase is a structural protein with a low copy number (31, 32). This could be the reason why we did not identify the ATPase in the HCIV-1 virion. However, this suggests that HCIV-1 could package its linear dsDNA molecule into a preformed procapsid also, as shown for PRD1 (31).

HCIV-1 sequence elements were also identified in some haloarchaeal chromosomes. The *Halobiforma lacisalsi* and *Haladaptatus paucihalophilus* chromosomal sequence elements known as proviruses HaloLacP1 and HalaPauP1 are related to alphasphaerolipoviruses (9, 10) and also share sequence similarities with HCIV-1 (see Table S5 in the supplemental material). In addition, we identified an HCIV-1-related region in *Haladaptatus cibarius* (NZ_JDTH01000002.1; 488,665 to 496,650) and here designated it provirus HalaCibP1 (see Table S5). HCIV-1-like elements in HaloLacP1, HalaPauP1, and HalaCibP1 cover ~54%, ~44%, and ~24%, respectively, of the HCIV-1 coding regions, including the genes encoding the MCPs and the ORF for putative packaging ATPase. A set of HCIV-1-related sequence elements, including the regions encoding the putative packaging ATPase and spike complex proteins VP3 and VP6, was also identified in *Natrialba aegyptia*, *Natrinema versiforme*, *Natrialba asiatica*, *Natrinema* sp. strain J7-2, *Halomicrobium mukohataei* (proviruses HaloMukP1 and HaloMukP2), and *Haloterrigena thermotolerans* genomes (see Table S5).

### Genome organization suggests that HCIV-1 is closely related to alphasphaerolipoviruses.

The overall levels of nucleotide identity of the HCIV-1 genome with the genomes of SH1, PH1, and HHIV-2 are ~63%, ~58%, and ~57%, respectively, and all of these viruses share overall gene synteny (Fig. 4C; see also Fig. S5 in the supplemental material). Only six HCIV-1 ORFs showed no similarity to SH1, PH1, or HHIV-2. However, ORF 4 and genes *27* (VP9), *28* (VP3), and *29* (VP6) had homologues only in the SH1 and PH1 genomes and not in HHIV-2. In SH1, proteins VP9, VP3, and VP6 are known to locate in the virion vertices (5), whereas HHIV-2 has divergent proteins for such host recognition structures (9, 24). There are also some other HCIV-1 ORFs that have a counterpart in only one virus or two viruses but not in all three alphasphaerolipoviruses: (i) HCIV-1 ORFs 11, 45, and 46 were highly similar to the corresponding ORFs only in SH1 but showed no similarity to PH1 or HHIV-2 sequences; (ii) HCIV-1 ORF 15 was similar only to HHIV-2 ORF 10; and (iii) HCIV-1 ORFs 31 and 43 had homologues only in PH1. In addition, the HCIV-1 ORF 41 homologue in the SH1, PH1, and HHIV-2 genomes exists as two adjacent ORFs.

Interestingly, while HCIV-1 protein VP2 (encoded by gene *24*) showed ~50% overall similarity to VP2 proteins of SH1, PH1, and HHIV-2 (see Table S2 in the supplemental material), the sequences flanking the heptapeptide repeats were more highly conserved (up to 93% amino acid similarity; Fig. 4D), suggesting that the function and interactions within the virion could be highly conserved. Similarly, although the HCIV-1 VP1 protein (gene *9*) showed only ~30% overall similarity to the SH1 VP1 protein (see Table S2), a distinct region in the C-terminal end of HCIV-1 VP1 (amino acids 1398 to 1608) was more conserved (~90% similarity; Fig. 4D).

Among the HCIV-1 structural proteins, those most conserved with respect to the group of SH1, HHIV-2, and PH1 were MCPs VP4 and VP7 (similarities, 86% to 94%) and the VP12 membrane protein (similarities, 93% to 96%) (see Table S2 and Fig. S5 in the supplemental material). These three form the core structural elements of the virion and are encoded by three adjacent genes.

**FIG 6:**
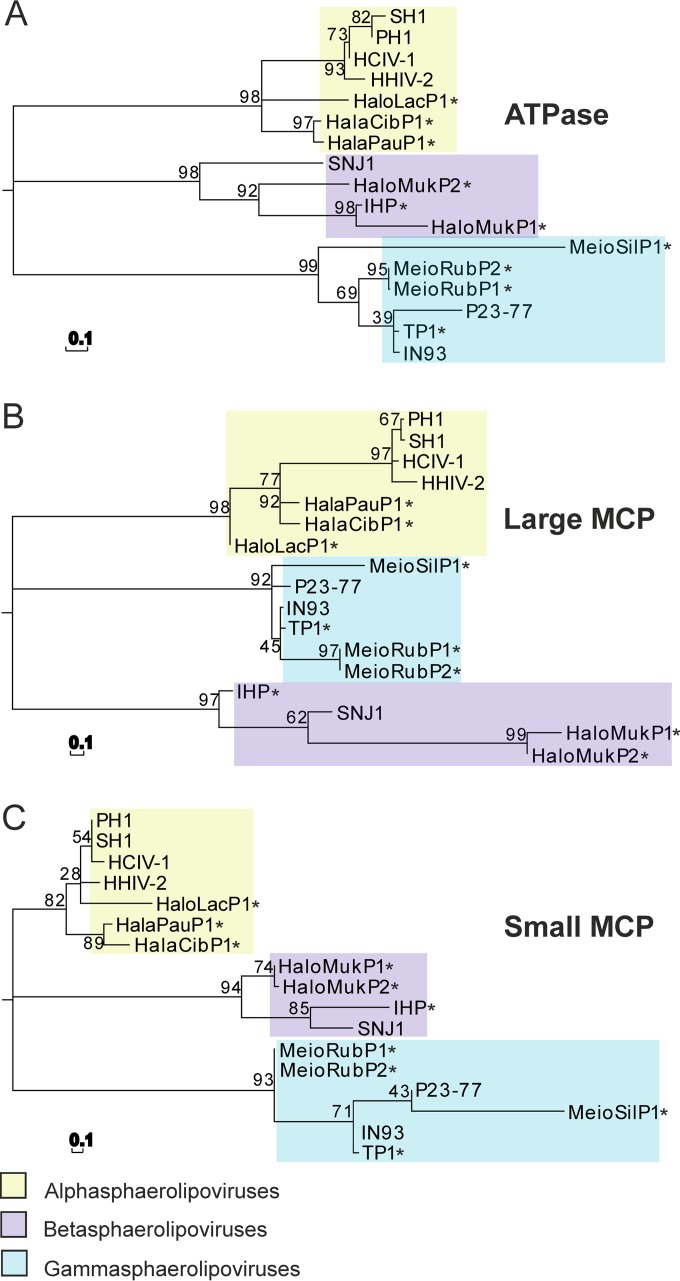
Phylogenetic analysis of conserved HCIV-1 proteins. Maximum likelihood phylogenetic trees of protein sequences of HCIV-1, sphaerolipoviruses, and related proviruses (marked with asterisks) are shown. (A) Putative ATPase. (B) Large MCP (VP4 in HCIV-1). (C) Small MCP (VP7 in HCIV-1). Evolutionary analysis was conducted using the JTT amino acid substitution model and 1,000 bootstrap values in MEGA 5.05. The bar (0.1) indicates the inferred number of substitutions per site.

In HHIV-2, the conserved cysteine residues capable of making disulfide bridges between the VP4 and VP7 MCPs are known to stabilize the capsid lattice (24). The VP4 of HCIV-1 lacks cysteine residues, whereas a conserved cysteine is present in MCP VP4 of HHIV-2 (Cys42), SH1, and PH1. However, the other conserved cysteine (Cys78 in HHIV-2 VP7) was identified in the HCIV-1 MCP VP7 and is also present in all other alphasphaerolipoviruses. The putative packaging ATPases of these viruses were also highly (93% to 97%) similar. The lowest similarities were seen among putative vertex complex proteins VP1 (32% to 35%), VP2 (47% to 53%), VP3 (50% to 54%), and VP6 (about 38% to 40%) (see Table S2 and Fig. S5 in the supplemental material), the last two of which were absent from HHIV-2 (9). The bootstrap consensus tree constructed on the basis of the sequences of MCPs and ATPases (Fig. 6) indicated that HCIV-1 clusters with the alphasphaerolipovirus clade.

## DISCUSSION

### Archaeal halophilic icosahedral internal membrane-containing sphaerolipoviruses.

Due to the short history of archaeal virus research, available information about tailless, icosahedral haloarchaeal viruses is limited. However, the marked increase in the availability of information during the recent years allows us to now make detailed comparisons (33). Here, we introduce HCIV-1, the most recent haloarchaeal tailless virus isolated from salt crystals (11). Phylogenetic reconstruction based on the sequences of the conserved structural elements of the icosahedral haloarchaeal viruses, thermophilic bacteriophages, and corresponding proviruses revealed that HCIV-1 clusters with SH1, HHIV-2, and PH1 into a distinct, well-supported clade (Fig. 6) (34). Consequently, we propose that HCIV-1 is a member of the *Alphasphaerolipovirus* genus of the *Sphaerolipoviridae* family. Here we identified one more HCIV-1-related provirus, HalaCibP1, in the genome of *Haladaptatus cibarius* (see Table S5 in the supplemental material), indicating that these viruses could be common in nature.

### HCIV-1 entry.

HCIV-1 isolated on *Haloarcula californiae* has a relatively narrow host range, infecting three additional euryarchaeal strains of 44 tested (10 genera) (11). A narrow host range is typical for the characterized halophilic icosahedral viruses (11, 35). In most cases, neither the receptor molecules nor the receptor recognition structures used by haloviruses to adsorb to host cells have been identified. However, it was shown that the 5-fold spike complexes of SH1 and HHIV-2, which are probably involved in host recognition and attachment, are completely different (5, 24). HCIV-1 contains homologues of SH1 major spike complex proteins VP3 and VP6, suggesting that the HCIV-1 vertex complexes are horn-shaped, like those of SH1 (5), although their host specificities differ (see Table S2 in the supplemental material). Interestingly, the HCIV-1 genes encoding VP3 and VP6 were also identified in the chromosomes of several haloarchaea (see Table S5), which suggests that this complex might have a cellular origin. The HCIV-1, SH1, and HHIV-2 adsorption rates fall in the same range (see Fig. S2A in the supplemental material) (9) but are lower than those of bacteriophages, possibly reflecting adaptation to the relatively low growth rate of archaea (36). Even the halophilic phages have higher adsorption rates than the haloarchaeal viruses (21, 36). HCIV-1 adsorption was most rapid during the first hour, but the maximal number of particles was bound to host cells 4 h later (see Fig. S2A), by which point some progeny virus particles had already assembled inside the cells. Thus, HCIV-1 infection was relatively nonsynchronous.

Early in the infection, TEM revealed tube-like structures between attached HCIV-1 particles and host cells (see Fig. 1B and C in the supplemental material). To our knowledge, structures of this form had not been previously reported for any archaeal virus. These structures might serve as virus genome delivery devices, as has been shown for those formed by tailless icosahedral bacteriophages PRD1 (37) and ɸX174 (38). To form these delivery conduits, PRD1 transforms its internal membrane into a tail-tube structure upon receptor binding (37), whereas ɸX174 oligomerizes its DNA pilot H protein (38).

### HCIV-1 progeny virion release.

To gain insight into the HCIV-1 virion release mechanism, we applied a combination of traditional metrics and potentiometry (Fig. 1). An electrochemical approach had been used previously to analyze life cycles of bacteriophages (39, 40), and it was recently adapted to the study of virus release under high-salinity conditions (27). The timing of cell lysis can be reliably determined by measuring the amount of ATP released from the cells and the level of oxygen consumption in the culture, combined with detection of PCB^−^ binding to the ruptured cell membranes (27). In the HCIV-1 single-step growth experiments, no decrease in cell culture turbidity was observed until ~12 h p.i. (Fig. 1A and G), while both progeny virus counts and biochemical measurements suggested that virus release begins earlier. As early as 10 h p.i., cell membranes were compromised, respiration had decreased, and ATP release from the cells had begun (Fig. 1F to I). The accumulation of cell debris at the same time indicated cell lysis (see Fig. S2B in the supplemental material). However, we could not identify any candidate genes involved in HCIV-1 exit from the cell. We assume that, due to the low adsorption rate and nonsynchronous infection, cell culture turbidity measurements are not sufficiently sensitive to detect cell lysis until the number of disrupted cells has become substantial. Moreover, since cell culture turbidity *per se* cannot verify cell viability, relying on turbidity measurements to determine the viral life cycle mode can be misleading, as was shown for virulent *Sulfolobus* virus SIRV2 (41). Currently, no archaeal virus lysis genes similar to those of bacteriophages have been recognized. Whether this observation reflects the difference between the cell envelope properties of archaea and bacteria remains to be seen (42). The only archaeal virus exit strategy described thus far is that of the STIV and SIRV2 release occurring through pyramid-like openings observed on the surface of *Sulfolobus* cells—a mechanism not described for any bacteriophage (43).

### HCIV-1 virion assembly model.

The HCIV-1 virion is icosahedral and consists of proteins, lipids, and an ~31-kb linear dsDNA molecule. On the basis of the high similarity of the amino acid sequences of the core structural proteins to those in the sphaerolipovirus group (Fig. 4; see also Table S2 in the supplemental material) (6, 7, 9, 24, 34), we propose an HCIV-1 virion assembly model that mimics the one suggested for HHIV-2 (24). In addition, on the basis of the similar diameters of the SH1, HHIV-2, and HCIV-1 virions, we can propose that the HCIV-1 capsid lattice geometry also corresponds to a pseudotriangulation number of T=28 (5, 24). In all PRD1-like viruses with vertical single β-barrel MCPs known to date, two adjacent genes encode the two MCPs (24). This arrangement is also found in the HCIV-1 genome (genes *20* and *21* coding for MCPs VP7 and VP4, respectively). On the basis of the model, the building blocks used for the formation of the HCIV-1 capsid lattice are homodimers of VP4 proteins and heterodimers of VP4 and VP7. The single β-barrels of VP7 and VP4 (with only one single β-barrel) form the lattice, whereas the second β-barrel of VP4 resides on top of the lattice-forming β-barrel and makes the turret on the capsomer surface. This arrangement was previously shown for HHIV-2 (24). The assembly of membrane-containing icosahedral viruses with two vertical single β-barrel MCPs is most probably aided by interactions between the MCPs and membrane proteins. Likewise, the conserved major membrane proteins of HCIV-1 (VP10 and VP12; Fig. 4) might offer a platform for correct capsomer assembly. During the assembly, viral lipids are selectively acquired from the host cytoplasmic membrane (Fig. 3). The HHIV-2 capsid is stabilized by disulfide bridges across the MCPs (24), and the same conserved cysteine residues are also present in the MCPs of SH1 and PH1 (VP4 Cys42 and VP7 Cys78). Interestingly, HCIV-1 VP7 contains the conserved Cys78, but the expected cysteine residue is not present in HCIV-1 VP4. This suggests that the linking between the MCPs could be different in HCIV-1 and that some other stabilizing mechanisms compensate for the lack of disulfide bridges. The spike complexes of HCIV-1 located at the 5-fold vertices of the icosahedral capsid are composed of at least proteins VP3 and VP6 and likely function in host receptor recognition.

### Conserved virion elements.

Notably, the putative MCPs as well as the putative packaging ATPase are highly conserved (over 90% similarity) among the alphasphaerolipoviruses, including HCIV-1. At the same time, the least conserved structural elements in this virus group are the proteins of the spike complex. This observation is in line with the idea that the hallmark of any viral group is their specific virion structure (12, 14, 44). Conservation of the pseudohexameric capsomer footprint was recently shown across a group of PRD1-like viruses with two vertical single β-barrel MCPs (HHIV-2, SH1, and P23-77), in contrast to their diverse host recognition structures (24). On the basis of the overall capsid organization, as well as the conserved sequence motif specific for packaging enzymes (15) present in its putative packaging ATPase, we suggest that HCIV-1 belongs to the PRD1-adenovirus structure-based lineage together with other alphasphaerolipoviruses. Strikingly, all viruses with single β-barrel MCPs known to date have been isolated from extreme environments.

### An astronomical number of viruses but only a few viral morphotypes.

Due to the astronomical number of viruses in the environment (45), the probability of isolating highly similar viruses twice should be practically zero. Nevertheless, it is remarkable that closely related viruses can be sampled from distinct locations all over the biosphere. The structural constraints and the limited number of protein folds restrict the diversity of virion morphotypes (14). What is known about the sphaerolipoviruses, including HCIV-1, is in accord with this observation. Another example is provided by the eight archaeal pleolipoviruses (46). Although they were isolated from distant geographical locations and at different times, they clearly resemble each other in their life cycles, structural protein and lipid profiles, and overall virion organization. These eight viruses carry genomes of different types, but they nevertheless share a cluster of conserved genes (46). Yet another example is provided by archaeal myoviruses HSTV-2, HF1, and HF2 (47, 48). Similar situations are known also for bacteriophages. Tectiviruses PRD1, PR3, PR4, PR5, L17, and PR772 have been isolated from different geographical locations but share a virion architecture and remarkable sequence identity at the nucleotide level (49). On the other hand, two subgroups of *Tectiviridae*, the temperate Bam35-like and the virulent PRD1-like viruses, do not have detectable sequence similarity, although their virion architectural principles are the same (50). It is evident that protein folds are more conserved over evolutionary time periods than the genomic or even protein sequences. Thus, related viruses with similar virion architectures that are distributed worldwide might nevertheless have no detectable sequence similarities. Therefore, the astronomical viral diversity resides at the sequence level, while the variety of virion architectures is highly limited.

## MATERIALS AND METHODS

### Viruses, archaeal strains, and growth conditions.

HCIV-1, SH1 (6), HHIV-2 (9), and HRPV-1 (51), as well as *Haloarcula californiae* ATCC 44799 (52), *Haloarcula hispanica* ATCC 33960 (53), and *Halorubrum* sp. strain PV6 (51), were grown aerobically at 37°C in modified growth media (MGM) containing artificial SW (47, 54). Broth and solid and top-layer agar plates contained 23% (wt/vol), 20%, and 18% SW, respectively. SH1, HHIV-2, and HRPV-1 were produced and purified as previously described (6, 9, 51). HCIV-1 virus stocks were prepared similarly, using semiconfluent plates.

To optimize virus production in liquid, *Haloarcula californiae* cells at the early (optical density at 550 nm [OD_550_], 0.3; ~1 × 10^7^ CFU/ml)-, middle (OD_550_, 0.6; ~1 × 10^8^ CFU/ml)-, and late (OD_550_, 0.9; ~2 × 10^8^ CFU/ml)-exponential-growth phases were infected with the virus stock at a MOI of 25. For optimization, cells (OD_550_, 0.6) were infected at different MOIs (3 to 30).

### Stability assays.

The infectivity of HCIV-1 samples was determined by plaque assay following the specified treatments. For determinations of sensitivity to lowered ionic strength, the virus stock was diluted 1,000-fold in 0.7% to 23% SW buffers and then incubated for 3 h and 24 h at 4°C. For determinations of the effects of NaCl and Mg^2+^, the virus stock was diluted in 23% SW devoid of NaCl or Mg^2+^ and then incubated as described above. For determinations of Ca^2+^ requirements, the virus stock was diluted 1,000-fold in 23% SW devoid of CaCl_2_ and then incubated 24 h at 4°C. For determinations of stability at 4°C, HCIV-1 was monitored for 2 weeks at 4°C in the HCIV-1 buffer (1 M NaCl, 70 mM MgCl_2_, 20 mM KCl, 1 mM CaCl_2_, 50 mM Tris-HCl [pH 7.2]; 1,000-fold dilution of the virus stock in the buffer). For determinations of temperature sensitivity, virus stock was incubated for 30 min at different temperatures (4°C to 90°C). For determinations of pH sensitivity, virus stock was diluted 1,000-fold in modified 23% SW containing 61 mM potassium phosphate (pH 3.8, 4.6, or 5.5) or 61 mM bis-Tris (pH 6.4 or 7.1) or 61 mM Tris-HCl (pH 8.0 or 9.0) and then incubated for 3 h and 24 h at 4°C.

### Adsorption assay and virus life cycle and electrochemical and ATP measurements during virus infection.

To determine the adsorption rate constant of HCIV-1, *Haloarcula californiae* cells (OD_550_, 0.6) were infected at a MOI of ~2 × 10^−3^ and then incubated aerobically at 37°C as previously described (9, 55). HCIV-1 infection was monitored in temperature-controlled 50-ml reaction vessels as described previously (27). *Haloarcula californiae* cells (OD_550_, 0.6; 30 ml) were infected at a MOI of 10 and incubated aerobically at 37°C. A similarly treated but noninfected culture was used as a control. The numbers of infective centers and viable cells were determined during the first 5 h p.i. Unadsorbed viruses were removed at 5 h p.i. by washing the cultures three times. The incubation was continued in vessels, and the turbidity of the cultures (OD_550_) was monitored. To determine the number of infectious progeny viruses in the medium, samples withdrawn from the infected culture starting at 6 h p.i. were centrifuged (Eppendorf 5415D centrifuge; 15,800 × *g*, 3 min, 22°C) and the supernatant was analyzed by plaque assay. Light microscopy analysis of the noninfected and infected cells (nondiluted samples) was performed using ×400 and ×1,000 magnification, oil immersion, and an Olympus BX50F-3 optical microscope equipped with a SensiCam cooled digital 12-bit charge-coupled-device (CCD) camera.

The concentration of PCB^−^ was monitored in the medium by selective electrodes using Ag/AgCl reference electrodes (Sigma-Aldrich) as described previously (27, 39). The PCB^−^ used was synthesized and purified by Aldona Beganskienė, Vilnius University, Lithuania. Heat-disrupted cells used as a control for PCB^−^ measurements (maximal PCB^−^ binding) were prepared by incubating the cells (OD_550_, 0.8) in boiling water for 10 min. The dissolved oxygen level in the medium was monitored by the use of selective electrodes (Orion model 9708; Thermo Scientific), and the amount of extracellular ATP in the medium was measured as described previously (27).

### Virus production and purification of HCIV-1.

*Haloarcula californiae* liquid cultures (OD_550_, 0.6) were infected at a MOI of 10. At ~50 h p.i., the cell debris was removed (BioSeal F12 rotor; 10,900 × *g*, 30 min, 4°C). Particles were precipitated from the supernatant using 10% (wt/vol) polyethylene glycol (PEG) 6000, collected (BioSeal F12 rotor; 10,900 × *g*, 40 min, 4°C), and resuspended in 18% SW (100-fold concentration). Aggregates were removed (Eppendorf 5415D; 15,800 × *g*, 10 min, 4°C), and the specimen was subjected to rate-zonal centrifugation in a linear 5% to 20% (wt/vol) sucrose gradient–18% SW (Sorvall AH629 rotor; 103,600 × *g*, 75 min, 15°C). The viral zone (once-purified HCIV-1) was collected and further purified by equilibrium centrifugation in a CsCl gradient in 18% SW (mean density of 1.3 g/ml) (Sorvall AH629 rotor; 72,000 × *g*, 16 to 20 h, 20°C). The virus zone (twice-purified HCIV-1) was diluted 2-fold with 18% SW without NaCl, concentrated by differential centrifugation (Sorvall T647.5 rotor; 113,600 × *g*, 3 h, 20°C), and resuspended in HCIV-1 buffer. For further analyses, CsCl equilibration centrifugation was used (Sorvall TH641 rotor; 209,600 × *g*, 27 h, 20°C).

### Electron microscopy.

For thin-section TEM, the *Haloarcula californiae* cells (OD_550_, 0.6) were infected by HCIV-1 (MOI of 10 or 60). Collected cells (Eppendorf 5415D; 2,300 × *g*, 5 min, 4°C) were resuspended in the original volume of MGM buffered with 61 mM 4-morpholineethane-sulfonic acid (pH 6.7), fixed with 3% (wt/vol) glutaraldehyde (20 min, 22°C), and washed three times with the same medium. Thin sections were prepared as previously described (56). For negative staining, the twice-purified HCIV-1 was stained with 3% (wt/vol) uranyl acetate (pH 4.5) for 30 s. Electron micrographs were taken with a Jeol 1400 electron microscope (Electron Microscopy Unit, Institute of Biotechnology, University of Helsinki) (80 kV).

### Protein and lipid analyses.

Protein concentrations were measured using bovine serum albumin as a standard (57). Tricine sodium dodecyl sulfate (SDS) polyacrylamide gels with 14% (wt/vol) and 4% (wt/vol) polyacrylamide in separation and stacking gels, respectively, were used (58). Coomassie brilliant blue R 250 (Serva; proteins), Sudan Black B (Sigma Aldrich; lipids), or pro-Q Emerald (Invitrogen; glycoproteins) and Sypro-Ruby (Invitrogen; proteins) were used for gel staining.

For N-terminal sequencing by Edman degradation (Procise Protein Sequencing System; Applied Biosystems, Life Technologies), proteins were separated in gels, transferred onto a polyvinyl difluoride membrane (ProBlott; Applied Biosystems, Life Technologies), and stained with Coomassie brilliant blue R 350 (GE Healthcare). For mass spectrometry, protein bands from the gels stained with Coomassie brilliant blue R 250 (Serva) were subjected to in-gel digestion (56). Cysteine bonds were reduced with dithiothreitol (Sigma Aldrich) and alkylated with iodoacetamide (Sigma Aldrich). After trypsinization (V5111; Promega) (sequencing-grade modified trypsin), peptides were quenched with 10% trifluoroacetic acid (TFA) and purified with C_18_ microspin columns (Harvard Apparatus), eluting the samples to 0.1% TFA–50% acetonitrile. The dried peptides were reconstituted in 0.1% TFA–1% acetonitrile (buffer A). Mass spectrometry analysis was carried out with an Orbitrap Elite ETD mass spectrometer (Thermo, Fisher Scientific) coupled with Proxeon Easy-nLC (Thermo, Fisher Scientific). The separation gradient included 5% buffer B (0.1% TFA–98% acetonitrile) processed for 5 min, 35% buffer B processed for 60 min, 80% buffer B processed for 5 min, and 100% buffer B processed for 10 min. Data were acquired with LTQ Tune software. Protein identification was carried at the Proteomics Unit, Institute of Biotechnology, University of Helsinki.

Lipids were extracted from twice-purified HCIV-1 and *Haloarcula californiae* and *Haloarcula hispanica* early-stationary-phase cells as previously described (59). Lipids stored in chloroform-methanol (9:1 [vol/vol]) were separated by thin-layer chromatography on silica gel 60 plates (Merck) with chloroform–methanol–90% acetic acid (65:4:35 [vol/vol/vol]) used as the solvent (60). Phospholipids were visualized by ammonium molybdate staining as described previously (61).

### Genome sequencing and analysis.

For nucleic acid extraction, the twice-purified HCIV-1 was treated with proteinase K (Thermo Scientific) (0.5 µg/ml) and 2% (wt/vol) SDS (45 min, 37°C). Phenol-ether-extracted nucleic acid was precipitated with NaCl and ethanol. To obtain the genome with possible terminal proteins, proteinase K was omitted from the reaction mixture. Purified nucleic acid was treated with RQ1 DNase (Promega), RNase A (Fermentas), Exonuclease III (Fermentas), Mung Bean nuclease (Promega), and restriction enzymes AseI, MseI, SspI, SalI, and HincII (New England Biolabs), as well as with SmaI and NotI (Fermentas). The genome was sequenced via the use of a shotgun library and subsequent Sanger sequencing (LGC Genomics, Berlin, Germany). After quality clipping (PHRED20) and vector clipping of the raw data, the program gap4 (Staden package; Roger Staden, Cambridge, United Kingdom) was used for data assembly. The 192 reads assembled into 4 contigs that provided 5.4× average coverage of the entire genome. Gaps were closed by PCR. In addition, terminal genome regions were sequenced directly on the genomic DNA toward the ends using a protocol optimized for large constructs (99 sequencing cycles) with several primers. The cycle sequencing was performed with a BigDye Terminator v3.1 cycle sequencing kit. Sequencing runs were executed using an ABI3730 XL sequencer and POP7 polymer.

The sequence was analyzed using Geneious version 6.1.6 by Biomatters (62). ORFs were predicted using GenMarkS (63) and Glimmer 3 (64). For the final ORF coordinates, preference was given to longer ORFs and to those displaying colinearity with previously annotated virus genomes. The percentage of GC content of predicted ORFs was calculated using Science Buddies’ “Genomics %G~C Content Calculator” (http://www.sciencebuddies.org/science-fair-projects/project_ideas/Genom_GC_Calculator.shtml). Isoelectric points and molecular masses of putative ORF products were calculated using the Compute pI/MW tool (65) of Expasy tools. tRNA sequences were searched with ARAGORN v1.2.36 (66). Overall nucleotide similarities between the genomes were calculated with EMBOSS Stretcher (67). Functions of predicted ORFs and putative conserved domains were analyzed by similarity searching (BLASTX and BLASTP) (68) against the NCBI nonredundant protein database (accessed 21 March 2016). For predictions of transmembrane helices and coiled-coil regions in proteins, the TMHMM v 2.0 server (69) and COILS tool (70) were used. Tandem and inverted repeats were searched using the Tandem Repeats Finder program (71) and EMBOSS Einverted (72). The nucleotide identities and amino acid similarities were calculated using EMBOSS Needle (67). Graphical visualization of amino acid similarities between viral putative proteins was done using Circos software (73).

Phylogenetic analysis of conserved protein sequences of HCIV-1, SH1, PH1, HHIV-2, SNJ1, IN93, P23-77, HaloLacP1 (10), HalaPauP1 (10), HalaCibP1 (in *Haladaptatus cibarius* NZ_JDTH01000002.1; 488,665 to 496,650), HaloMukP1 (23), HalomukP2 (23), MeioRubP1 (23), MeioRubP2 (34), MeioSilP1 (23), IHP (22), and TP1 (34) was conducted using Molecular Evolutionary Genetics Analysis (MEGA) software (version 5.05) (74). The sequences (NCBI database) were aligned using the MUSCLE program (75), and the tree was built using a maximum likelihood method, the Jones-Taylor-Thornton (JTT) amino acid substitution model, and 1,000 bootstrap values.

### Accession number.

The HCIV-1 genome sequence has been deposited in the NCBI database under accession number KT809302.

## SUPPLEMENTAL MATERIAL

Figure S1  Stability of HCIV-1 virions. (A) Infectivity in SW with decreasing ionic strength (squares, NaCl concentration) determined after 3 h (closed circles) and 24 h (open circles). (B) Infectivity in SW adjusted to the specified NaCl concentrations. (C) Infectivity in SW adjusted to the specified Mg^2+^ concentrations. (D) Stability of infectivity for the virus stock (open circles) and virus in HCIV-1 buffer (closed circles). The infectivity scales (left *y* axis) are the same for panels A to D. (E) Infectivity after 30 min of incubation at different temperatures. (F) Infectivity of the virus after 30 min (closed circles) and 24 h (open circles) of incubation in modified 23% SW of different pHs. The infectivity scales (left *y* axis) are the same for panels E and F. Download Figure S1, TIF file, 0.6 MB

Figure S2  (A) Adsorption of HCIV-1 to *Haloarcula californiae* cells at 37°C. Error bars represent standard deviations. (B) Light microscopy of uninfected and HCIV-1-infected *Haloarcula californiae* cells (MOI of 10) cultured at 37°C with aeration in the reaction vessels. In both cultures, cells were washed at 5 h p.i., which removed the unadsorbed virus particles from the infected culture. Cell debris is circled. Undiluted samples, ×1,000 magnification (oil immersion). Download Figure S2, PDF file, 1.6 MB

Figure S3  HCIV-1 structural proteins analyzed in polyacrylamide-Tricine-SDS gel stained with pro-Q Emerald 300 (for glycoproteins only) (A) and SYPRO-Ruby (for proteins) (B). CandyCane molecular mass standards (in kilodaltons) (M; left lane), including both glycosylated and nonglycosylated proteins, are indicated. Major HRPV-1 and HCIV-1 virion proteins (right), including glycosylated HRPV-1VP4, are shown. Download Figure S3, TIF file, 0.1 MB

Figure S4  HCIV-1 protein VP2 heptapeptide repeat (DXAARGY, where X is D/E and Y is A/S/T). The HCIV-1 VP2 repeat region (with numbered amino acids residues 225 to 456) is represented by the aligned repeat units. Download Figure S4, TIF file, 1.9 MB

Figure S5  Significant (>55%) amino acid similarity between HCIV-1 ORFs/gene products and those of SH1, PH1, and HHIV-2. Similar ORF/gene products in different viruses are connected. ORFs/genes, light grey; noncoding regions, dark grey; genome reading directions, arrows (see Fig. 4). ORFs or genes coding for virion proteins (VPs) are indicated. Download Figure S5, PDF file, 0.2 MB

Table S1  Recovery of infectious particles during HCIV-1 purification. Table S1, PDF file, 0.01 MB

Table S2  HCIV-1 ORFs and genes. Table S2, PDF file, 0.2 MB

Table S3  Repeats in HCIV-1 genome. Table S3, PDF file, 0.02 MB

Table S4  Conserved domains in predicted HCIV-1 proteins. Table S4, PDF file, 0.04 MB

Table S5  HCIV-1-related elements in archaeal genomes. Table S5, PDF file, 0.03 MB
