# Supplementary figures and images for: Archaeal Haloarcula californiae Icosahedral Virus 1 Highlights Conserved Elements in Icosahedral Membrane-Containing DNA Viruses from Extreme Environments
Source: mBio. 2016 Jul 19;7(4):e00699-16. doi: 10.1128/mBio.00699-16 (PMC4958249; doi:10.1128/mBio.00699-16)

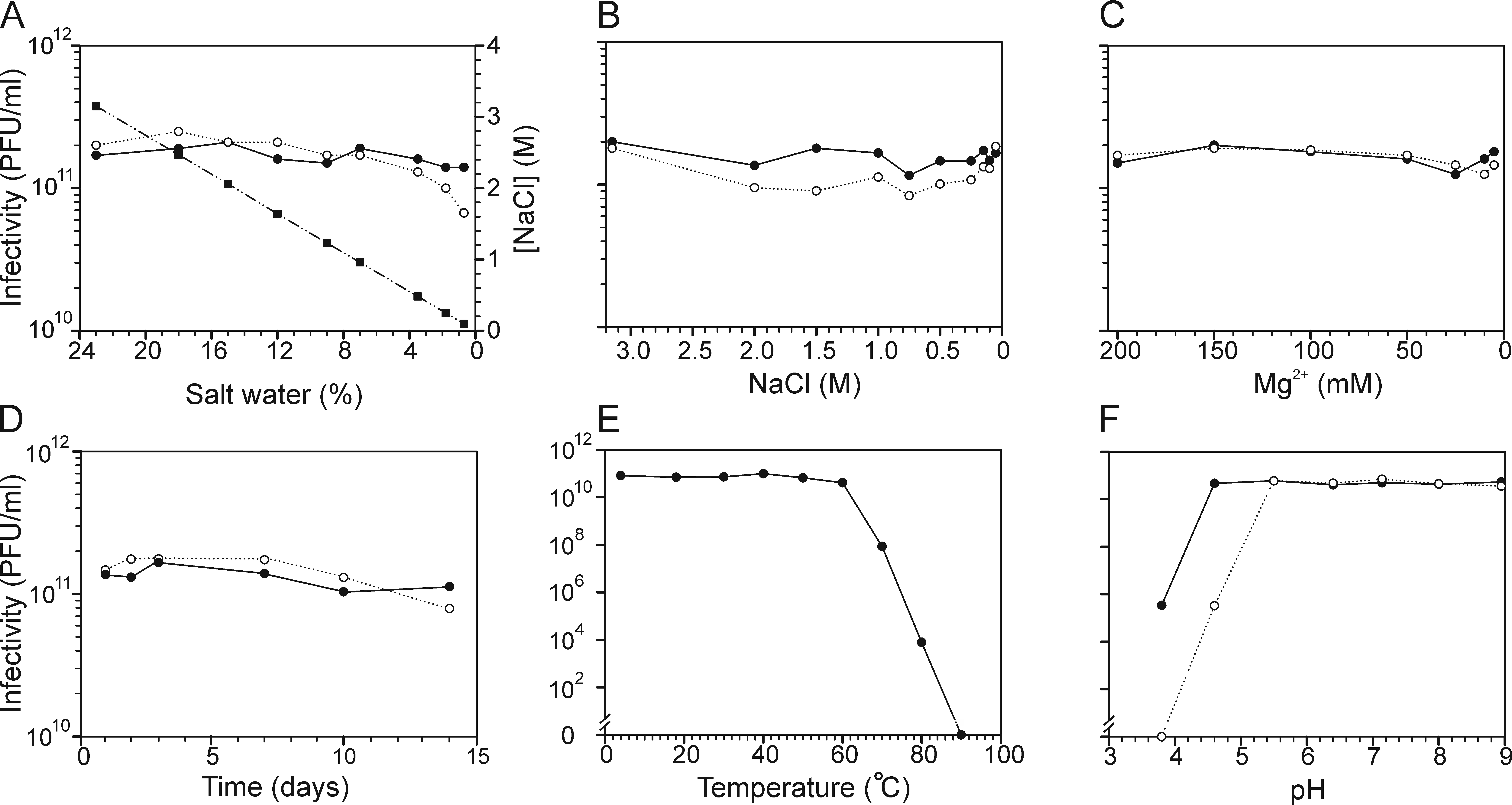

Supplement: Figure S1 — Stability of HCIV-1 virions. (A) Infectivity in SW with decreasing ionic strength (squares, NaCl concentration) determined after 3 h (closed circles) and 24 h (open circles). (B) Infectivity in SW adjusted to the specified NaCl concentrations. (C) Infectivity in SW adjusted to the specified Mg2+ concentrations. (D) Stability of infectivity for the virus stock (open circles) and virus in HCIV-1 buffer (closed circles). The infectivity scales (left y axis) are the same for panels A to D. (E) Infectivity after 30 min of incubation at different temperatures. (F) Infectivity of the virus after 30 min (closed circles) and 24 h (open circles) of incubation in modified 23% SW of different pHs. The infectivity scales (left y axis) are the same for panels E and F. Download [file mbo004162905sf1.tif]

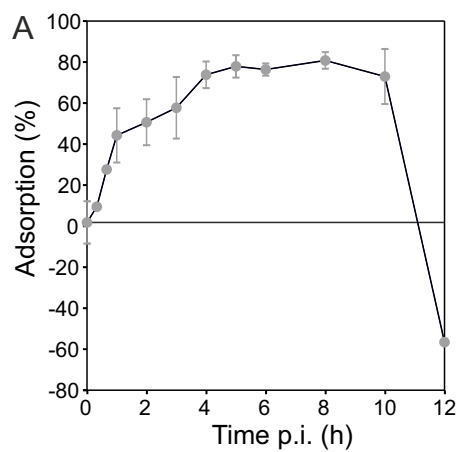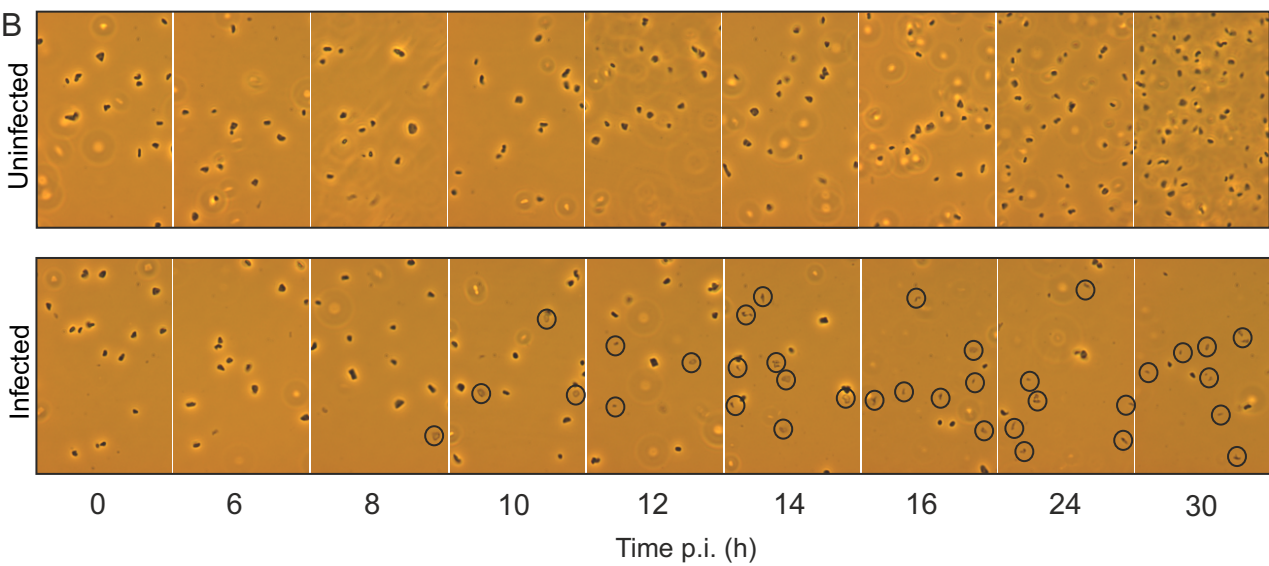

Supplement: Figure S2 — (A) Adsorption of HCIV-1 to Haloarcula californiae cells at 37°C. Error bars represent standard deviations. (B) Light microscopy of uninfected and HCIV-1-infected Haloarcula californiae cells (MOI of 10) cultured at 37°C with aeration in the reaction vessels. In both cultures, cells were washed at 5 h p.i., which removed the unadsorbed virus particles from the infected culture. Cell debris is circled. Undiluted samples, ×1,000 magnification (oil immersion). Download [file mbo004162905sf2.pdf]

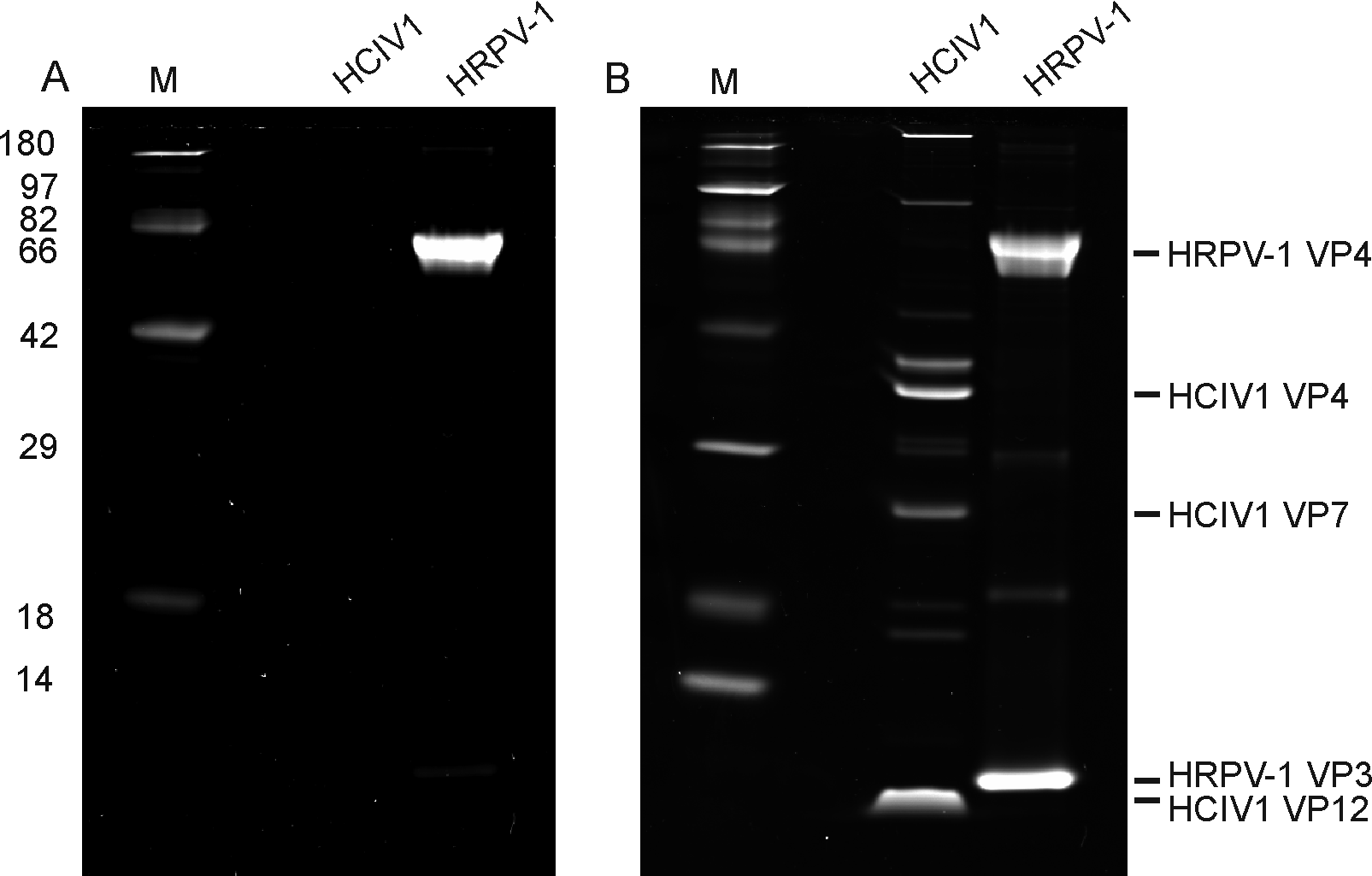

Supplement: Figure S3 — HCIV-1 structural proteins analyzed in polyacrylamide-Tricine-SDS gel stained with pro-Q Emerald 300 (for glycoproteins only) (A) and SYPRO-Ruby (for proteins) (B). CandyCane molecular mass standards (in kilodaltons) (M; left lane), including both glycosylated and nonglycosylated proteins, are indicated. Major HRPV-1 and HCIV-1 virion proteins (right), including glycosylated HRPV-1VP4, are shown. Download [file mbo004162905sf3.tif]

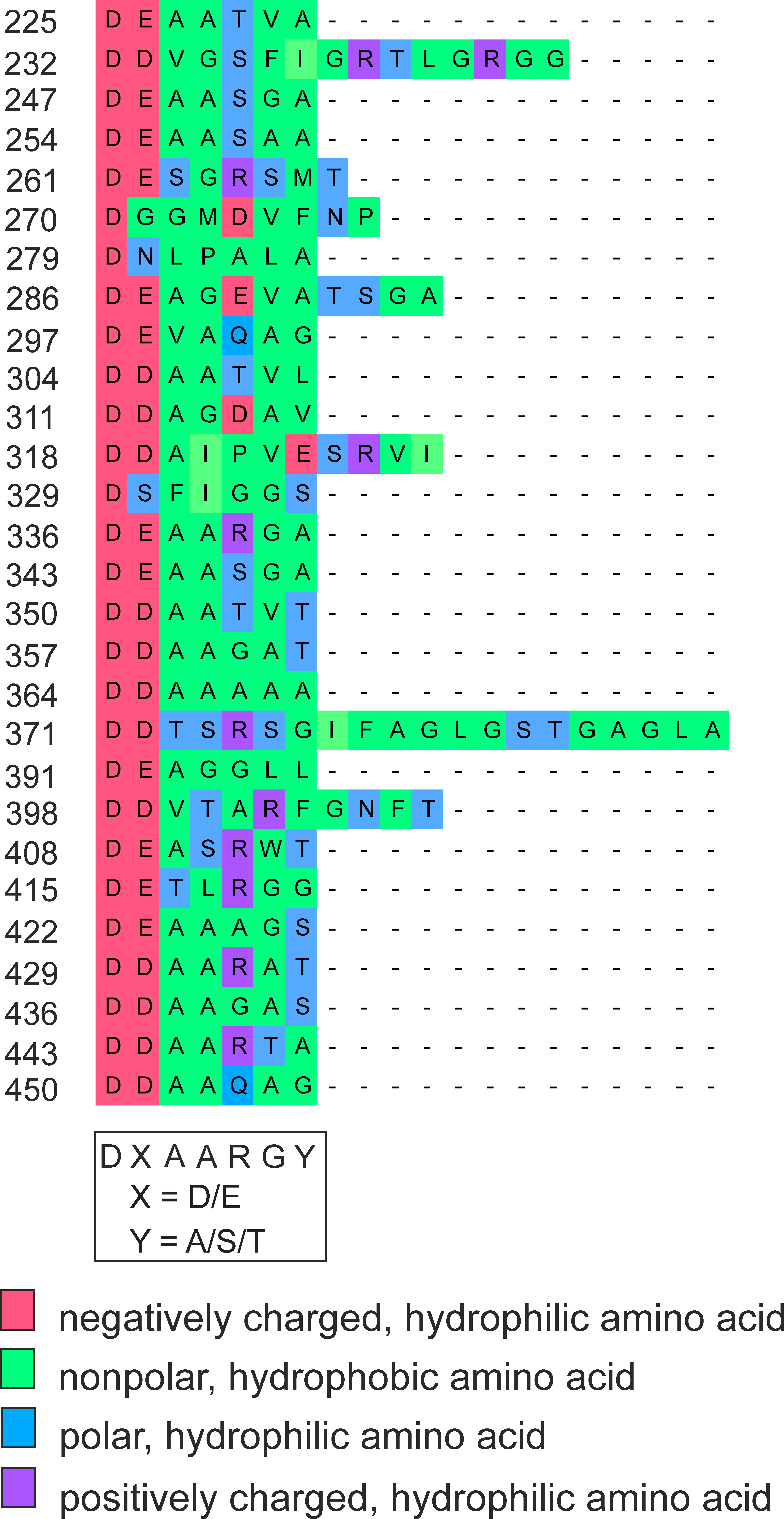

Supplement: Figure S4 — HCIV-1 protein VP2 heptapeptide repeat (DXAARGY, where X is D/E and Y is A/S/T). The HCIV-1 VP2 repeat region (with numbered amino acids residues 225 to 456) is represented by the aligned repeat units. Download [file mbo004162905sf4.tif]
