# Supplementary material for: Archaeal Haloarcula californiae Icosahedral Virus 1 Highlights Conserved Elements in Icosahedral Membrane-Containing DNA Viruses from Extreme Environments
Source: mBio. 2016 Jul 19;7(4):e00699-16. doi: 10.1128/mBio.00699-16 (PMC4958249; doi:10.1128/mBio.00699-16)
Supplement: Figure S5 — Significant (>55%) amino acid similarity between HCIV-1 ORFs/gene products and those of SH1, PH1, and HHIV-2. Similar ORF/gene products in different viruses are connected. ORFs/genes, light grey; noncoding regions, dark grey; genome reading directions, arrows (see Fig. 4). ORFs or genes coding for virion proteins (VPs) are indicated. Download [file mbo004162905sf5.pdf]

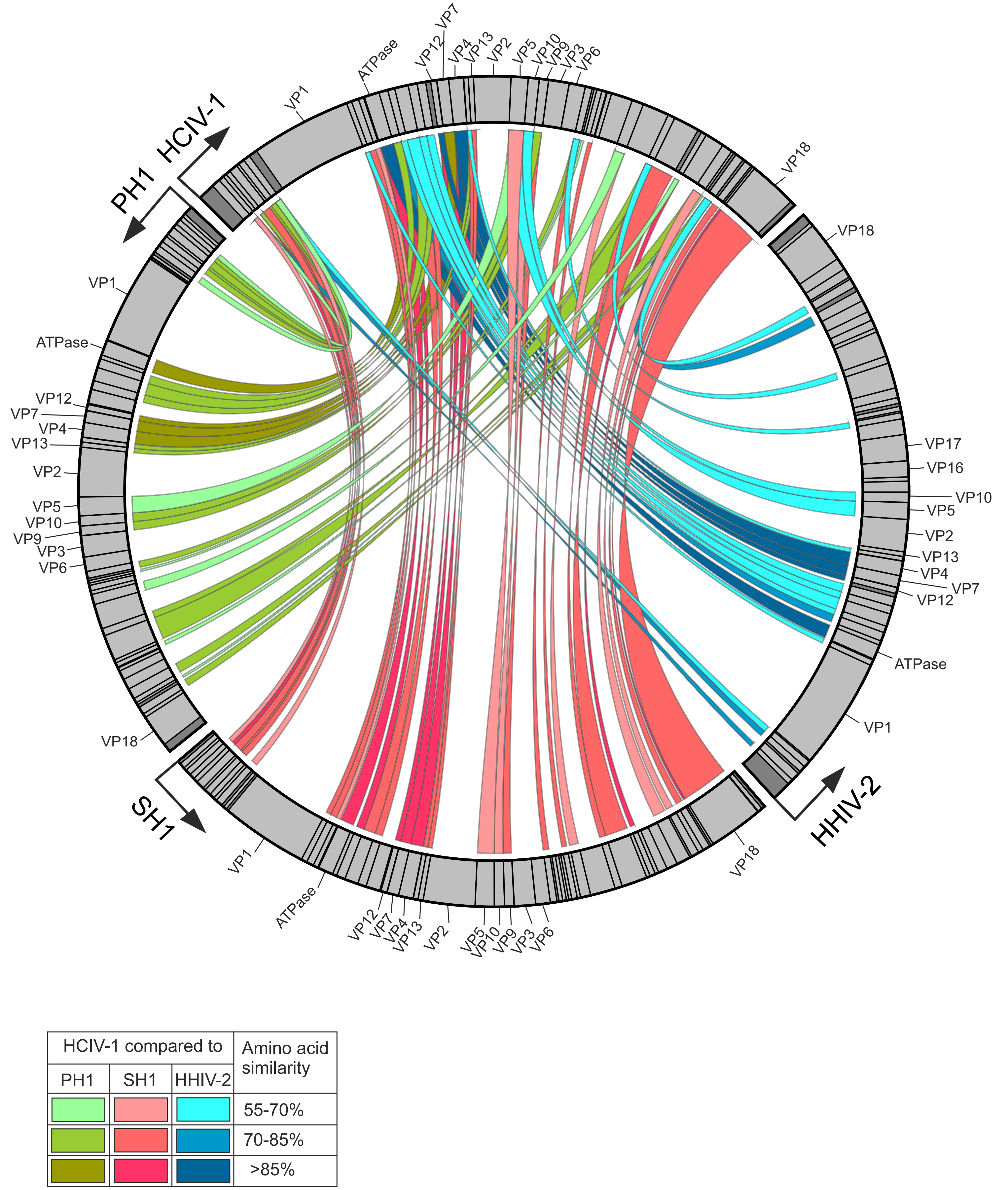
HCIV-1
PH1
SH1
HHIV-2
VP1
ATPase
VP12
VP7
VP4
VP13
VP2
VP5
VP10
VP9
VP3
VP6
VP18
VP17
VP16
HCIV-1 compared to
PH1
SH1
HHIV-2
Amino acid similarity
55-70%
70-85%
>85%
