# Supplementary material for: Archaeal Haloarcula californiae Icosahedral Virus 1 Highlights Conserved Elements in Icosahedral Membrane-Containing DNA Viruses from Extreme Environments
Source: mBio. 2016 Jul 19;7(4):e00699-16. doi: 10.1128/mBio.00699-16 (PMC4958249; doi:10.1128/mBio.00699-16)
Supplement: Table S1 — Recovery of infectious particles during HCIV-1 purification. [file mbo004162905st1.pdf]

TABLE S1. Recovery of infectious particles during HCIV-1 purification.

Purification step	Titer (PFU/ml)[a]	Total PFUs[b]	Recovery of infectivity (%)
Cell lysate	1.4×10^{10}	1.4×10^{13}	100.0
PEG-NaCl precipitate	1.1×10^{12}	1.0×10^{13}	71.4
Purified viral zone in sucrose gradient	1.3×10^{11}	6.2×10^{12}	44.3
Purified viral zone in CsCl gradient	1.6×10^{11}	4.8×10^{12}	34.5
Concentrate (twice-purified virus)	1.8×10^{13}	2.4×10^{12}	17.2

[a] Average of six independent experiments.

[b] Calculated per liter of culture.
