# Supplementary material for: Archaeal Haloarcula californiae Icosahedral Virus 1 Highlights Conserved Elements in Icosahedral Membrane-Containing DNA Viruses from Extreme Environments
Source: mBio. 2016 Jul 19;7(4):e00699-16. doi: 10.1128/mBio.00699-16 (PMC4958249; doi:10.1128/mBio.00699-16)
Supplement: Table S2 — HCIV-1 ORFs and genes. [file mbo004162905st2.pdf]

TABLE S2. HCIV-1 ORFs and genes.

ORF/ gene	Protein	Cons. domains[a]	Direction[b]	Start[c]	Stop[c]	GC (%)	Residues	Calc. MW (kDa)	Calc. pI	TMH[d]	Coils[e]	Similar viral (putative) proteins[f]	Amino acid similarity (%)
ORF 1	putative protein 1		F	815	1234	72.9	140	14.93	4.45			HSTV-2 hypothetical protein HSTV2 69	43.3
												HRTV-7 hypothetical protein HRTV7 67	43.3
												HRTV-5 hypothetical protein HRTV5 78	44.4
												HRTV-8 hypothetical protein HRTV8 80	38.4
												HF2 hypothetical protein HF2p037	38.7
ORF 2	putative protein 2		F	1235	1483	68.3	83	9.01	4.30			HHIV-2 putative protein 1	70.6
												SH1 ORF 4 product	61.2
												PH1 hypothetical protein HhPH1_gp03	58.8
ORF 3	putative protein 3		F	1485	1664	66.1	60	6.82	11.20	1		-	
ORF 4	putative protein 4		F	1661	1888	68	76	8.62	3.90			SH1 ORF 5 product	88.0
												PH1 hypothetical protein HhPH1_gp04	80.0
ORF 5	putative protein 5		F	1881	2300	71.2	140	14.51	4.75	2		SH1 ORF 6 product	78.1
												PH1 hypothetical protein HhPH1_gp05	73.0
												HHIV-2 putative protein 2	48.7
ORF 6	putative protein 6		F	2297	2434	71.7	46	4.84	8.19		+	-	
ORF 7	putative protein 7		F	2427	2735	68.3	103	11.72	4.59			SH1 ORF 8 product	84.6
												PH1 hypothetical protein HhPH1_gp07	84.3
												HHIV-2 putative protein 3	72.9
												HHTV-2 hypothetical protein HHTV2 26	41.7
												Mycobacterium phage Dori gp40	41.7
												Bacillus phage G gp248	41.0
												Bacillus phage vB BanS-Tsamsa hypothetical protein gp17	36.2
												Streptomyces phage Jay2Jay hypothetical protein PBI_JAY2JAY_122	35.0
												Streptomyces phage VWB hypothetical protein VWBp17	30.1

												Streptomyces phage SF3 hypothetical protein SF3_590	28.6
ORF 8	putative protein 8		F	2728	3054	68.8	109	12.57	4.91			SH1 ORF 11 product	67.5
												PH1 hypothetical protein HhPH1_gp09	67.8
												HHIV-2 putative protein 4	65.5
												SNJ1 ORF 17 product	40.0
												Bacillus phage Shanette hypothetical protein SHANETTE_61	38.7
gene *9*	VP1	+	F	3472	8298	68.3	1609	176.87	4.23		+	SH1 VP1	34.7
												PH1 capsid protein VP1	31.9
												HHIV-2 VP1	32.2
ORF 10	putative protein 10		F	8300	8548	70.7	83	8.55	5.64	2		SH1 ORF 14 product	76.1
												HHIV-2 putative protein 6	64.4
												HSTV-1 hypothetical protein HSTV1 16	41.0
ORF 11	putative protein 11		F	8545	8898	70.9	118	12.07	4.41	3		SH1 ORF 15 product	83.8
												His1 hypothetical protein His1V_gp13	44.1
												HHTV-2 hypothetical protein HHTV2_54	38.4
ORF 12	putative protein 12		F	8891	9139	67.1	83	9.42	4.14			SH1 ORF 16 product	62.4
ORF 13	putative ATPase	+	R	9913	9191	63.8	241	26.93	5.6			PH1 putative ATPase	97.1
												SH1 ORF 17 product (putative ATPase)	96.2
												HHIV-2 putative ATPase	92.9
												SNJ1 putative ATPase	37.4
ORF 14	putative protein 14		R	10345	9914	66.2	144	15.46	4.32		+	SH1 ORF 19 product	87.7
												PH1 hypothetical protein HhPH1_gp15	84.4
												HHIV-2 putative protein 9	84.6
ORF 15	putative protein 15		R	10679	10338	67.5	114	12.27	4.84			HHIV-2 putative protein 10	58.7
ORF 16	putative protein 16		R	11332	10676	68.5	219	22.84	3.33		+	SH1 ORF 20 product	81.5
												PH1 hypothetical protein HhPH1_gp16	80.1
												HHIV-2 putative protein 11	68.9

ORF 17	putative protein 17	R	11757	11332	67.4	142	14.46	3.81			PH1 hypothetical protein HhPH1_gp17	79.9
											SH1 ORF 21	80.9
											HHIV-2 putative protein 12	60.6
ORF 18	putative protein 18	R	12172	11762	67.6	137	14.88	3.97			PH1 hypothetical protein HhPH1_gp18	48.4
											HHIV-2 putative protein 13	64.2
											SH1 ORF 22 product	46.3
gene *19*	VP12	F	12412	12696	60.7	95	9.81	10.44	2		SH1 VP12	95.7
											HHIV-2 VP 12	92.6
											PH1 capsid protein VP 12	93.6
gene *20*	VP7	F	12712	13226	63.6	185	19.89	4.38			PH1 capsid protein VP 7	93.5
											SH1 VP7	93.5
											HHIV-2 VP 7	85.9
											SNJ1 PB6	32.4
gene *21*	VP4	F	13269	13967	65.8	233	25.98	4.14			PH1 capsid protein VP 4	92.2
											SH1 VP4	93.1
											HHIV-2 VP4	90.1
											SNJ1 PB2	29.0
ORF 22	putative protein 22	F	13983	14198	63	72	7.65	3.71			PH1 hypothetical protein HhPH1_gp22	79.5
											SH1 ORF 26 product	76.7
											HHIV-2 putative protein 17	60.6
ORF 23	VP13[g]	F	14202	14447	66.7	82	8.80	4.61			PH1 capsid protein VP13	81.5
											SH1 VP13	80.2
											HHIV-2 VP 13	54.8
gene *24*	VP2	F	14449	16230	73.6	594	58.16	3.8		+	SH1 VP2	47.5
											PH1 VP2	47.4
											HHIV-2 VP2	52.7
gene *25*	VP5	F	16230	17045	69.9	272	28.57	4.33			SH1 VP5	69.6
											PH1 capsid protein VP 5	69.0

												HHIV-2 VP 5	58.9
gene *26*	VP10		F	17048	17569	67.8	174	17.68	4.33		+	PH1 capsid protein VP 10	76.0
												SH1 VP10	65.9
												HHIV-2 VP 10	57.3
gene *27*	VP9		F	17572	18012	60.3	147	16.25	4.65			SH1 VP9	78.9
												PH1 VP 9	77.0
												SNJ1 ORF 1	31.0
gene *28*	VP3		F	18018	18998	61.7	327	35.67	4.11			PH1 capsid protein VP3	54.2
												SH1 VP3	50.8
gene *29*	VP6		F	19005	19781	64.1	259	28.07	4.23			PH1 capsid protein VP6	40.3
												SH1 VP6	37.9
ORF 30	putative protein 30		F	19781	20110	68.8	110	11.77	4.90	1		SH1 ORF 34 product	77.7
												PH1 hypothetical protein HhPH1	74.8
												HHIV-2 putative protein 27	58.6
												HCTV-2 hypothetical protein HCTV2 61	27.6
ORF 31	putative protein 31		R	20339	20208	68.2	44	5.03	4.7			PH1 hypothetical protein HhPH1_gp31	67.4
ORF 32	putative protein 32	+	R	20587	20336	71.8	84	9.25	4.7			-	
ORF 33	putative protein 33		R	20810	20589	67.6	74	7.91	4.78			SH1 ORF 39 product	71.9
												PH1 hypothetical protein HhPH1_gp33	49.5
												HHIV-2 putative protein 29	45.7
ORF 34	putative protein 34	+	R	21063	20848	67.6	72	7.95	4.48			-	
ORF 35	putative protein 35		R	22091	21060	65.2	344	40.15	4.85		+	Pseudomonas phage PaMx25 putative DNA topoisomerase	41.2
												Roseobacter phage RDJL Phi 2 hypothetical protein RDJLphi2_gp23	40.1
												Cellulophaga phage phi3ST:2 hypothetical protein phi3ST:2 gp62	39.5
												Xylella phage Sano hypothetical protein Sano 37	38.4
												Salmonella phage BP12C hypothetical protein BP12C_28	38.4

											Xylella phage Salvo hypothetical protein Salvo 33	36.3
											Salmonella phage SPN19 hypothetical protein SPN19 026	36.1
											Roseobacter phage RDJL Phi 1 hypothetical protein RDJLphi1_gp26	28.5
ORF 36	putative protein 36		R	22648	22088	69.9	187	20.52	6.05		PH1 hypothetical protein HhPH1_gp35	57.9
											SH1 ORF 41 product	58.1
											HHIV-2 putative protein 30	53.7
ORF 37	putative protein 37		R	23919	22645	68.5	425	46.80	4.66		SH1 ORF 42 product	50.0
											PH1 hypothetical protein HhPH1_gp36	49.8
											HHIV-2 putative protein 31	46.5
ORF 38	putative protein 38		R	24277	23921	70.3	119	12.59	4.29		PH1 hypothetical protein HhPH1_gp37	72.4
											SH1 ORF 43 product	75.6
											HHIV-2 putative protein 32	52.3
ORF 39	putative protein 39		R	25505	24261	68.5	415	46.36	4.36	+	SH1 ORF 44 product	82.3
											PH1 hypothetical protein HhPH1_gp38	80.0
											HHIV-2 putative protein 33	55.1
ORF 40	putative protein 40		R	25956	25699	64.3	86	9.98	4.45		SH1 ORF 46 product	91.8
											HHIV-2 putative protein 35	47.8
											PH1 hypothetical protein HhPH1_gp40	68.2
ORF 41	putative protein 41	+	R	26882	25959	70.3	308	35.01	4.6	+	SH1 ORF 48 product	38.8
											PH1 hypothetical protein HhPH1_gp42	38.8
											HHIV-2 putative protein 36	35.5
											SH1 ORF 49 product	40.4
											PH1 hypothetical protein HhPH1_gp43	37.9
											HHIV-2 putative protein 37	36.8
ORF 42	putative protein 42	+	R	27394	26879	69	172	18.77	4.55		PH1 hypothetical protein HhPH1_gp44	81.7
											SH1 ORF 50 product	64.3
											HHIV-2 putative protein 38	77.0

											Halorubrum pleomorphic virus 3 ORF 6 product	31.2
											HHTV-1 hypothetical protein HHTV1 72	27.9
ORF 43	putative protein 43		R	27507	27391	65.8	39	4.06	4.3		PH1 hypothetical protein HhPH1_gp46	65.8
ORF 44	putative protein 44	+	R	28050	27643	74.3	136	14.48	4.27	+	PH1 hypothetical protein HhPH1_gp47	74.6
											SH1 ORF 51 product	67.4
											HHIV-2 putative protein 39	58.8
											Environmental halophage eHP-31 hypothetical protein OSG_eHP31_00085	27.6
ORF 45	putative protein 45		R	28528	28178	71.2	117	12.83	4.04		SH1 ORF 53 product	78.8
ORF 46	putative protein 46	+	R	28737	28624	70.2	38	3.92	7.69		SH1 ORF 54 product	86.5
ORF 47	VP18[g]		R	31055	28734	72.5	774	84.71	4.16	+	SH1 ORF 55 product	77.3
											PH1 VP18	33.9
											HHIV-2 VP18	33.8

[a] Predicted conserved domains, for detailed information about conserved domains see Table S5.

[b] F, forward (transcribed from left to right in the linear genome); R, reverse (transcribed from right to left in the linear genome).

[c] Nucleotide coordinates refer to the HCIV-1 genome sequence.

[d] Number of transmembrane helices (TMH).

[e] +, ORF/gene products with predicted coil-coiled region(s).

[f] BLAST search dated 21 March 2016. (Putative) proteins with ≥25 % amino acid similarity to HCIV-1 sequences are listed.

[g] Assignment is based on the sequence similarities with the corresponding sequences in SH1, PH1, and HHIV-2.
