# Supplementary material for: Archaeal Haloarcula californiae Icosahedral Virus 1 Highlights Conserved Elements in Icosahedral Membrane-Containing DNA Viruses from Extreme Environments
Source: mBio. 2016 Jul 19;7(4):e00699-16. doi: 10.1128/mBio.00699-16 (PMC4958249; doi:10.1128/mBio.00699-16)
Supplement: Table S3 — Repeats in HCIV-1 genome. [file mbo004162905st3.pdf]

TABLE S3. Repeats in HCIV-1 genome.

Tandem repeats[a]

Coordinates[b]	Consensus sequence (size in nt)	Copy number	Match (%)
11044-11088 (ORF 16)	cgtcgccgccgtcgtcac (18)	2.5	96
15186-15239 (ORF 24)	cgacgaggcggccagcgccgc (21)	2.5	81
15449-15503 (ORF 24)	ccgacgaggcggccacgcggcg (22)	2.6	80
15754-15803 (ORF 24)	gacgacgctgcacggaccgct (21)	2.4	87
30127-30156 (ORF 47)	aggtcgtcggcctcc (15)	2.0	93
30492-30533 (ORF 47)	gtcgtcgtcgcc (12)	3.5	87
31197-31243 (right end of the genome)	gcggtccgaccggcccct (18)	2.6	80

Inverted repeats[c]

Coordinates[b]	Sequence[d]	Match (%)
11047-11087/16326-16286 (ORF 16/ORF 25)	cgtcgccgtcgtcaccgtcgccgccgtcgtcaccgtcgccg	90
446-475/20161-20190 (Upstream ORF 1/ between ORF 30 and ORF 31)	aggcgcacagacggcccgtgagcggttccg	96
15296-15394/29492- 29590 (ORF 24/ORF 47)	tggccgacgaggccggcgaggtcg-caaccagcggggccgacga ggtggcccaggccggcgacgacgccgccaccgtgctggacgacg- ccgggacgccg	75

[a] Tandem repeats found with Tandem Repeats Finder program.

[b] Nucleotide coordinates in HCIV-1 sequence.

[c] Inverted repeats found with Einverted program.

[d] Underlined, sequence mismatch; hyphen, gap in sequence.
