# Supplementary material for: Archaeal Haloarcula californiae Icosahedral Virus 1 Highlights Conserved Elements in Icosahedral Membrane-Containing DNA Viruses from Extreme Environments
Source: mBio. 2016 Jul 19;7(4):e00699-16. doi: 10.1128/mBio.00699-16 (PMC4958249; doi:10.1128/mBio.00699-16)
Supplement: Table S4 — Conserved domains in predicted HCIV-1 proteins. [file mbo004162905st4.pdf]

TABLE S4. Conserved domains in HCIV-1 predicted proteins.

HCIV-1 (putative) proteins	Domain hit[a]				
	Name	Accession	Description	Amino acid Coordinates	BLAST e-value
VP1	PCNA	cd00577	Proliferating Cell Nuclear Antigen (PCNA) domain found in eukaryotes and archaea	376-601	4.51e-20
	PCNA	cd00577	Proliferating Cell Nuclear Antigen (PCNA) domain found in eukaryotes and archaea	5-223	3.22e-10
	PRK01115	PRK01115	DNA polymerase sliding clamp	363-602	2.15e-31
	PRK01115	PRK01115	DNA polymerase sliding clamp	3-247	2.43e-09
	DnaN	COG0592	DNA polymerase sliding clamp subunit (PCNA homolog)	376-516	1.42e-04
	TIGR03491	TIGR03491	RecB family nuclease, putative, TM0106 family	91-159	6.96e-04
Putative ATPase	AAA_10	pfam12846	AAA-like domain	92-192	1.39e-07
	Yjgr	COG0433	Archaeal DNA helicase HerA or a related bacterial ATPase	119-211	2.51e-04
VP5	cpdB	PRK09420	Bifunctional 2',3'-cyclic nucleotide 2'-phosphodiesterase/3'-nucleotidase periplasmic precursor protein	167-264	9.99e-03
Putative protein 32	phenyl_P_alpha	TIGR02723	Phenylphosphate carboxylase, alpha subunit	7-50	2.69e-03
Putative protein 33	PRK14293	PRK14293	Chaperon protein DnaJ	38-71	7.25e-03
Putative protein 34	SIR2	COG0846	NAD-dependent protein deacetylases, SIR2 family	2-50	1.77e-03
	PRK06082	PRK06082	4-aminobutyrate aminotransferase	30-49	5.24e-03
	SIR2-fam	cd01407	Sir2 proteins (gene regulators)	10-50	9.46e-03
Putative protein 41	DUF4326	pfam14216	Domain of unknown function (DUF4326)	206-279	1.74e-10
	PTZ00121	PTZ00121	MAEBL	3-227	4.11e-04
Putative protein 42	TPMT	pfam05724	Thiopurine S-methyltransferase (TPMT)	83-164	2.04e-03
Putative protein 44	Sigma70_r4_2	pfam08281	Sigma-70, region 4	65-108	1.18e-07
	Sigma70_r4	cd06171	Sigma70, region (SR) 4	65-105	3.36e-05
	tfx	PRK03975	Putative transcriptional regulator	60-134	5.58e-09
	SigX5	TIGR02960	RNA polymerase sigma-70 factor, TIGR02960 family	32-127	2.28e-07
	RpoE	COG1595	DNA-directed RNA polymerase specialized sigma subunit, sigma24 homolog	10-105	3.08e-07
Putative protein 46	HypA	pfam01155	Hydrogenase expression/synthesis hypA family	6-37	3.00e-04
	hypA	PRK00564	Hydrogenase nickel incorporation protein	5-31	9.57e-03
	PriA	COG1198	Primosomal protein N' (replication factor Y) - superfamily II helicase	3-34	3.74e-05

[a] Hits were found with BLASTp program, search dated 21 March 2016 (e-value threshold 0.01).
