# Supplementary material for: Archaeal Haloarcula californiae Icosahedral Virus 1 Highlights Conserved Elements in Icosahedral Membrane-Containing DNA Viruses from Extreme Environments
Source: mBio. 2016 Jul 19;7(4):e00699-16. doi: 10.1128/mBio.00699-16 (PMC4958249; doi:10.1128/mBio.00699-16)
Supplement: Table S5 — HCIV-1-related elements in archaeal genomes. [file mbo004162905st5.pdf]

TABLE S5. HCIV-1-related elements in archaeal genomes.

| Archaeal species | HCIV-1 ORFs/genes[a] |
|---|---|
| *Halobiforma lacisalsi* (provirus HaloLacP1) | 5, 8, *9* (VP1), 11, 13 (putative ATPase, ~75%), 14, 16, 17, 18, *19* (VP12), *20* (VP7, ~66%), *21* (VP4, ~67%), 23, *24* (VP2), *25* (VP5), *26* (VP10), 42, 44 |
| *Haladaptatus paucihalophilus* (provirus HalaPauP1) | 8, *9* (VP1), 11, 13 (putative ATPase, ~76%), 14, 16, 18, *19* (VP12), *20* (VP7, ~70%), *21* (VP4, ~69%), *24* (VP2), *25* (VP5), *26* (VP10), *27* (VP9) |
| *Haladaptus cibarius* (provirus HalaCibP1) | 13 (putative ATPase, ~76%), 14, 16, 18, *20* (VP7, ~69%), *21* (VP4, ~67%), *24* (VP2), *25* (VP5), *26* (VP10), *27* (VP9) |
| *Natrialba aegyptia* | 8, 11, 13 (putative ATPase, ~33%), *28* (VP3), *29* (VP6), 42 |
| *Natrinema versiforme* | 8, 11, 13 (putative ATPase, ~31%), *28* (VP3), *29* (VP6), 42 |
| *Natrialba asiatica* | 8, 11, 13 (putative ATPase, ~33%), *29* (VP6), 42 |
| *Natrinema* sp. J7-2 | 8, 13 (putative ATPase, ~32%), *28* (VP3), *29* (VP6), 42 |
| *Halomicrobium mukahataei* | 11, 13 (putative ATPase, ~ 37% to provirus HaloMukP1 and ~ 29% to provirus HaloMukP2), *29* (VP6), 42 |
| *Haloterrigena thermotolerans* | 8, 13 (putative ATPase, ~32%), *28* (VP3), *29* (VP6), 42 |
| *Natrinema gari* | 8, 11, *28* (VP3), *29* (VP6) |
| *Haloterrigena jeotgali* | 8, *28* (VP3), *29* (VP6), 42 |

[a] HCIV-1 ORFs/genes with detected homologous proteins in archaeal genomes (search dated 21 March 2016). (%), amino acid similarities shown for conserved proteins (MCPs and ATPase).
